# Adult Human Primary Cardiomyocyte-Based Model for the Simultaneous Prediction of Drug-Induced Inotropic and Pro-arrhythmia Risk

**DOI:** 10.3389/fphys.2017.01073

**Published:** 2017-12-19

**Authors:** Nathalie Nguyen, William Nguyen, Brynna Nguyenton, Phachareeya Ratchada, Guy Page, Paul E. Miller, Andre Ghetti, Najah Abi-Gerges

**Affiliations:** AnaBios Corporation, San Diego, CA, United States

**Keywords:** human heart, adult human primary cardiomyocyte, pro-arrhythmia, inotropy, risk assessment, drug discovery and development

## Abstract

Cardiac safety remains the leading cause of drug development discontinuation. We developed a human cardiomyocyte-based model that has the potential to provide a predictive preclinical approach for simultaneously predicting drug-induced inotropic and pro-arrhythmia risk.

**Methods:** Adult human primary cardiomyocytes from ethically consented organ donors were used to measure contractility transients. We used measures of changes in contractility parameters as markers to infer both drug-induced inotropic effect (sarcomere shortening) and pro-arrhythmia (aftercontraction, AC); contractility escape (CE); time to 90% relaxation (TR90). We addressed the clinical relevance of this approach by evaluating the effects of 23 torsadogenic and 10 non-torsadogenic drugs. Each drug was tested separately at four multiples of the free effective therapeutic plasma concentration (fETPC).

**Results:** Human cardiomyocyte-based model differentiated between torsadogenic and non-torsadogenic drugs. For example, dofetilide, a torsadogenic drug, caused ACs and increased TR90 starting at 10-fold the fETPC, while CE events were observed at the highest multiple of fETPC (100-fold). Verapamil, a non-torsadogenic drug, did not change TR90 and induced no AC or CE up to the highest multiple of fETPCs tested in this study (222-fold). When drug pro-arrhythmic activity was evaluated at 10-fold of the fETPC, AC parameter had excellent assay sensitivity and specificity values of 96 and 100%, respectively. This high predictivity supports the translational safety potential of this preparation and of the selected marker. The data demonstrate that human cardiomyocytes could also identify drugs associated with inotropic effects. hERG channel blockers, like dofetilide, had no effects on sarcomere shortening, while multi-ion channel blockers, like verapamil, inhibited sarcomere shortening.

**Conclusions:** Isolated adult human primary cardiomyocytes can simultaneously predict risks associated with inotropic activity and pro-arrhythmia and may enable the generation of reliable and predictive data for assessing human cardiotoxicity at an early stage in drug discovery.

## Introduction

Cardiac safety remains the leading cause of drug development discontinuation and withdrawal of marketed drugs (Piccini et al., 2009). Consequently, during the last decade strategies have been extensively employed to evaluate the cardiac safety of novel drugs at the preclinical stage. However, the strategies employed thus far have proven to be prone to false positive signals, which may lead to prematurely discontinue the development of potentially useful drugs. In other cases, the occurrence of false negative results has led to serious adverse events during clinical trials. The limited predictivity of the current strategies has stimulated a quest for more reliable tools (Sager et al., 2014; Holmes et al., 2015; Gintant et al., 2017). Given that the challenges in translating preclinical findings into successful clinical studies, seem to originate, at least in part, from the use of animal models and the inability of different species to quantitatively recapitulate human cardiac physiology and pharmacology (Perel et al., 2007; Seok et al., 2013), the use of adult human cardiac tissue has the potential to provide the preclinical models needed to enhance preclinical to clinical translation. Human heart tissues and human isolated myocytes have been used for decades in *ex-vivo* studies of human physiology (see, for example, Bustamante et al., 1982; Beuckelmann et al., 1992; Wettwer et al., 1993, 1994; Näbauer et al., 1996; Iost et al., 1998; Näbauer and Kääb, 1998; Jost et al., 2005; Brandenburger et al., 2012; Coppini et al., 2014; Boukens et al., 2015). However, the adoption of these methods to drug discovery has been hampered by the limited availability of human tissue for research, the variability in the quality of the samples, and the technical challenges related to human tissue's procurement and experimental interrogation. For human cardiac tissue to have practical utility in preclinical cardiac safety assessment, it is necessary to develop and validate: (i) methodologies that can provide tissue of high and consistent quality; (ii) assays that can generate predictive data and are relatively simple and scalable to medium or high throughput format. To this aim, we have developed procedures that consistently allow the procurement and experimental interrogation of human heart tissue preparations to reliably assess the toxicity risks of novel drugs (Page et al., 2016). In order to further increase the throughput and scalability of the human *ex-vivo* heart model, we are now reporting on the implementation of a cell-based assay that utilizes adult human primary cardiomyocytes.

Regular heart beat and myocardial contractility (inotropy) are the essential properties of cardiac function and depend on the electro-mechanical dynamics of cardiac tissue. The consequence of drug-induced irregular heart beat (pro-arrhythmia; Sager et al., 2014) and/or changes in contractility (inotropic liability; Harmer et al., 2012; Gallacher et al., 2016; Pugsley et al., 2017) can limit the utility of potential novel therapeutic applications. Therefore, it is highly recommended to assess the potential of novel drugs to induce pro-arrhythmia and inotropic risk early in the drug discovery process before advancing into later development work.

Abnormal ventricular repolarization, such as the kind observed in patients with long QT syndrome, can cause not only electrical disorders (pro-arrhythmia) but also affect the heart's contractile function (Belardinelli et al., 2009; De Ferrari and Schartz, 2009). Long QT syndrome patients exhibit abnormal left ventricular contraction, which can appear as single or double-peaked contraction transient (Nador et al., 1991; De Ferrari et al., 1994), increased dispersion of myocardial contraction and abnormal left ventricular relaxation (Nakayama et al., 1998; Haugaa et al., 2009). This correlation between electrical (action potential, AP) and mechanical (contraction) abnormalities is a consequence of the tight functional coupling (Lou et al., 2011; Kang et al., 2016), and suggest that similarly to the genetic disorders which affect the QT interval, drug-induced ventricular repolarization abnormalities could lead to contractility changes. Along these lines, we investigated the possibility of developing a cardiomyocyte-based model that would allow the simultaneous evaluation of drug-related risks for pro-arrhythmia and inotropic liabilities. The main motivation of this investigation was to develop a cardiomyocyte-based model that uses adult human primary cardiomyocytes to provide a novel and predictive preclinical approach for the simultaneous prediction of drug-induced inotropic and pro-arrhythmia risk. In order to facilitate the scalability of the model, we focused on the simple measurement of a contractility-related parameter: we recorded the fractional sarcomere shortening, using a digital, cell geometry measurement system (IonOptix™; Abi-Gerges et al., 2013) and then used measures of changes in the contractility transients to infer both inotropic as well as pro-arrhythmia risk. To address the clinical relevance of this approach, we performed a validation study to test the effects of a set of 33 reference drugs with well-characterized clinical outcomes. Both positive and negative controls were selected, including 23 torsadogenic and 10 non-torsadogenic drugs. We found that the isolated cardiomyocytes accurately exhibited drug-induced contractility changes and pro-arrhythmia that are consistent with the known clinical safety profiles of the drugs tested.

## Materials and methods

### Donor heart procurement

All human hearts used for this study were non-transplantable and ethically obtained by legal consent (first person or next-of-kin) from organ donors in the United States. Our recovery protocols were pre-approved by IRBs at each transplant center. Furthermore, all transfers of the donor hearts are fully traceable and periodically reviewed by US Federal authorities. Donor characteristics are shown in Table 1 and exclusion criteria were previously described (Page et al., 2016).

**Table 1:** Donor characteristics.

**Heart no**.	**Donor identifier**	**Age**	**Sex**	**Ethnicity**	**BMI**	**COD**	**EF (%)**
1	160610HHA	26	F	Hispanic	26.9	Anoxia	65
2	161102HHA	39	M	Caucasian	22.5	CVA/ICH	60
3	161115HHA	27	M	Hispanic	25.9	Anoxia	60
4	161201HHA	37	F	Caucasian	29.1	Anoxia	65
5	170712HHB	29	F	Asian	21.9	Anoxia	N/A^a^
6	170815HHA	56	M	Caucasian	28.1	Head trauma	55
7	170822HHA	45	M	Hispanic	24.7	Head trauma	70
8	170906HHA	38	F	Caucasian	19.5	AS/Suicide	N/A^a^
9	170915HHA	21	M	Caucasian	32.0	Head trauma	65
10	171008HHA	45	M	Hispanic	21.1	CVA/Stroke	55
11	171025HHA	33	M	Hispanic	30.3	CVA/ICH	60

*F, Female; M, Male; BMI, Body Mass Index; COD, Cause Of Death; EF, Ejection Fraction; CVA, Cerebrovascular Accident; ICH, Intracranial Hemorrhage; AS, Asphyxiation; HH, Human Heart; HHA, the 1st heart received on the day; HHB, the 2nd heart received the same day*.

a *Organ procurement organization could not transplant the heart and consequently no echocardiography was performed; N/A, Not available*.

### Cardiomyocyte contractility measurement

Upon arrival at our laboratory, hearts were re-perfused with ice-cold proprietary cardioplegic solution as previously described (Page et al., 2016). Adult human primary ventricular myocytes were isolated enzymatically from the ventricles (Supplementary Figure 1). Digestion of the cardiac tissue was conducted at 37°C for ~25 min utilizing a proprietary solution which included a cocktail of proteolytic enzymes. Solutions and cells described in this paper will be available upon request. Contractility transients were measured as previously described (Harmer et al., 2012; Butler et al., 2015; Supplementary Video 1). Briefly, cardiomyocytes were placed in a perfusion chamber (FHC Inc., Bowdoin, ME, USA) mounted on the stage of an inverted Motic AE31E microscope (StellarScientific, MD, USA) and continuously perfused from a gravity fed system at 4 ml/min with myocyte Tyrode solution (see composition below) heated to ~36°C using an inline heater (Cell MicroControls, Norfolk, VA, USA). A video-based cell geometry system was used to measure sarcomere dynamics (IonOptix, MA, USA; Ren and Wold, 2001). The myocytes were field stimulated at voltage 50% above threshold at a 1 Hz pacing frequency, with a biphasic pulse of 3 ms duration, using a pair of platinum wires placed on opposite sides of the chamber and connected to a MyoPacer EP stimulator (IonOptix). Images were acquired at a rate of 240 Hz using an IonOptix MyoCam-S CCD camera. Digitized images were displayed within the IonWizard acquisition software (IonOptix). Optical intensity data were collected from a user-defined rectangular region of interest placed over the myocyte image. The optical intensity data represent the bright and dark bands corresponding to the Z-bands of the cardiomyocyte. The IonWizard software analyzes the periodicity in the optical density along the myocyte detecting the Z-bands by means of a fast Fourier transform algorithm.

The stability of sarcomere shortening transients was assessed by continuous recording for 2 min in Tyrode's solution establishing the vehicle control (in 0.1% dimethyl sulfoxide, DMSO). Subsequently, the test article concentration was applied for a minimum of 250 s period or until a steady-state effect was achieved. Four ascending concentrations of the test article were used, providing cumulative concentration-effect (C-E) curves. Analysis was performed using the IonWizard software. For each test condition, data for 15 contractions with or without AC or CE events were averaged, to obtain a single representative monotonic contractility transient. A series of polynomials were fitted to the five different phases of the monotonic transient. From this representative transient, fractional sarcomere shortening (which indicates the percentage of peak contraction relative to the resting length; μm) and TR90 (time to 90% relaxation; ms) were used to quantify sarcomere dynamics and delay in the relaxation of cardiomyocytes after contraction, respectively. An AC (after-contraction) was visually identified as change in the slope of the contractility transient that occurred before the next stimulus-induced contraction. CE (contractility escape) was also visually identified when the electrical stimulus did not result in a contraction transient. Presence or absence of AC and CE events was determined by examining non-averaged transients for the 4-min application article concentration. Results are expressed as mean ± s.e.m. Treatment effects on sarcomere shortening and TR90 were expressed relatively to the myocyte's specific baseline control period. AC and CE were expressed as incidence: number of cells showing events normalized by the total number of cardiomyocytes. Hill curves were fitted to sarcomere shortening C-E data using SigmaPlot v13 (Systat Software Inc., CA, USA) and used to determine IC_50_ (concentration inducing 50% decrease in sarcomere shortening). A comparative set of experiments were also performed with quinidine and verapamil on ventricular myocytes isolated from beagle dog hearts as previously described (Abi-Gerges et al., 2013). The dog beagle hearts were obtained from BTS Research (CA, USA) following the vendor's Institutional Review Board-approved protocols. Differences were tested for statistical significance using the paired Student's *t*-test. A value of *P* < 0.05 was considered significant.

Assessment of variability was assessed as previously described (Page et al., 2016). The intra-heart and inter-heart total variabilities were evaluated as follows. For baseline vehicle condition, the intra-heart variability for each parameter of the contractility transient was calculated as the average of the all SDs (Standard Deviations) generated from all the individual cells for all of the hearts. Inter-heart (Total) variability was calculated as the SD of all cells pooled at each parameter for all hearts. For dofetilide, the mean and SD of the percent change effect in the cells from each heart were calculated separately for each of the four test concentration periods. For each concentration period, the intra-heart variability was then calculated as the average of the all of the SDs generated from all the three individual hearts. Total variability was calculated as the SD of the mean percent change of all the cells pooled at each concentration period for dofetilide.

### Solutions and test articles

The standard myocyte Tyrode solution contained (in mM): NaCl 145, KCl 4, CaCl_2_ 1.8, MgCl_2_ 1, glucose 11.1 and HEPES 10, pH 7.4 with NaOH. The reference drugs selected for this investigation were obtained from Sigma (CA, USA). Drugs were initially formulated in DMSO as a 1,000x stock solution. Stock solutions were diluted to the working concentrations in 0.1% DMSO on the day of the experiment. The test concentrations are indicated in Table 2. Ratio to fETPCs (free Effective Therapeutic Plasma Concentration) and replicates information are also shown in Table 2.

**Table 2:** Concentrations tested in adult human primary cardiomyocyte-based model and ratio to clinical concentrations.

**Drug name**	**Clinical TdP risk**	**Cell/Heart (*n*)**	**fETPC (μM)^b^^,^^c^**	**Concentrations tested (**μ**M)**				**Concentrations as multiple of fETPC**			
Ajmaline		5/1	0.065	0.065	0.195	0.65	1.95	1	3	10	30
Astemizole^a^		4/1	0.0003	0.0003	0.0009	0.003	0.009	1	3	10	30
Azimilide^a^		6/1	0.07	0.07	0.21	0.7	2.1	1	3	10	30
Bepridil^a^		7/1	0.032	0.032	0.096	0.32	0.96	1	3	10	30
Chlorpromazine^a^		8/2	0.0345	0.0345	0.1035	0.345	1.035	1	3	10	30
Cisapride^a^		7/2	0.00258	0.00258	0.0258	0.0774	0.258	1	10	30	100
Clarithromycin^a^		8/2	1.2	1.2	12	36	120	1	10	30	100
Clozapine^a^		4/1	0.071	0.071	0.213	0.71	2.13	1	3	10	30
D,L-Sotalol^a^		8/2	14.7	1.5	15	150	450	0.1	1	10	30
Disopyramide^a^		7/2	0.7	0.7	2.1	7	21	1	3	10	30
Dofetilide^a^		6/1	0.002	0.002	0.02	0.06	0.22	1	10	30	100
Domperidone^a^		8/1	0.02	0.02	0.2	0.6	2	1	10	30	100
Droperidol^a^		4/1	0.016	0.016	0.048	0.16	0.48	1	3	10	30
Erythromycin		7/1	0.17	0.17	0.51	1.7	5.1	1	3	10	30
Flecainide		3/1	0.753	0.753	2.259	7.53	22.59	1	3	10	30
Ibutilide^a^		4/1	0.1	0.1	0.3	1	3	1	3	10	30
Moxifloxacin		5/1	10.96	10.96	32.88	109.6	328.8	1	3	10	30
Ondansetron^a^		4/1	0.372	0.372	1.116	3.72	11.16	1	3	10	30
Procainamide		4/1	54.186	54.186	162.558	541.86	1625.58	1	3	10	30
Quinidine^a^		4/1	3	0.3	3	30	100	0.1	1	10	30
Sematilide		6/1	4.449	4.449	13.347	44.49	133.47	1	3	10	30
Terodiline		4/1	0.145	0.145	0.435	1.45	4.35	1	3	10	30
Vandetanib^a^		4/1	0.3	0.3	0.9	3	9	1	3	10	30
Diltiazem^a^		4/1	0.128	0.128	0.384	1.28	3.84	1	3	10	30
Diphenhydramine		5/1	0.034	0.034	0.102	0.34	1.02	1	3	10	30
Loratidine^a^		4/1	0.00045	0.00045	0.00135	0.0045	0.0135	1	3	10	30
Mexiletine^a^		7/1	2.5	0.25	2.5	25	75	0.1	1	10	30
Mibefradil		6/1	0.012	0.012	0.036	0.12	0.36	1	3	10	30
Nifedipine^a^		4/1	0.0077	0.0077	0.0231	0.077	0.231	1	3	10	30
Nitrendipine^a^		4/1	0.00302	0.00302	0.00906	0.0302	0.0906	1	3	10	30
Ranolazine^a^		3/1	2	2	20	60	200	1	10	30	100
Tamoxifen^a^		6/1	0.0221	0.0221	0.0663	0.221	0.663	1	3	10	30
Verapamil^a^		4/1	0.045	0.01	0.1	1	10	0.2	2	22	222

a CiPA-selected drug;

b Redfern et al. (2003);

c *CiPA Stem Cell Working Group; TdP, Torsades de Pointes; fETPC, free Effective Therapeutic Plasma Concentration; Red, Positive pro-arrhythmia risk; Green, Negative pro-arrhythmia risk*.

## Results

In order to record contractility transients in isolated cardiomyocytes, we utilized bright-field optical imaging and measured sarcomere length. With the ultimate goal of assessing both electrical (AP) as well as mechanical (contractility) drug-induced effects, we decided to focus our analysis on four parameters: TR90, incidence of AC, incidence of CE and sarcomere shortening. TR90 is correlated to the duration of the cardiac AP and delays in AP repolarization are expected to be associated with extension of the TR90 (Dipla et al., 1999; Undrovinas et al., 2006). Early-afterdepolarization (EAD) is an AP abnormality that results in a transient slope change of the AP during the repolarization phase. EAD is potentially of great relevance in the context of pro-arrhythmia risk assessment since this is believed to be the underlying cause of re-entrant arrhythmia (Roden et al., 1996; El-Sherif and Turitto, 1999). The mechanical equivalent of EAD electrical abnormality is an after-contraction (Kaumann and Olson, 1968; Noda et al., 2004), a transient change of slope in the contractility transient, typically in the later portion of the relaxation phase. Drugs that interfere with cardiac depolarization or in other ways suppress the generation of a cardiac AP, result in complete inhibition of the contractility transient, an event we refer to as CE. Therefore, changes in three parameters measured from contractility transients, TR90, AC and CE can provide useful information with regards to the drug-induced alterations of the electrical behavior of cardiac cells. In addition, changes in fractional sarcomere shortening provide direct measurement of inotropic effects in cardiomyocytes.

### Stability of the contractility transient in adult human primary ventricular cardiomyocytes

Baseline properties of the contractility transients in adult human primary ventricular cardiomyocytes were investigated in 189 cardiomyocytes from 11 human donor hearts (Table 1). First, we calculated TR90, incidence of AC and CE, and sarcomere shortening in baseline vehicle control at a pacing rate of 1 Hz (Table 3). The distributions of the contractility parameters from all the vehicle baseline control periods show that at baseline, the physiological properties of isolated ventricular cardiomyocytes fall within the expected ranges (see section Discussion; Table 3; Supplementary Figure 2) with time to peak (TPeak) at 168 ± 3. ms and TR90 at 337 ± 8 ms. It is also important to note that during the vehicle baseline period, we never observed AC or CE events. We further assessed the intra-heart and inter-heart (Total) variability of contractility parameters in the presence of vehicle controls (Supplementary Figure 3). Our data showed that the intra-heart variability for sarcomere shortening, TPeak and TR70-90 accounted for almost 90% of the Total observed variability for each of these contractility transient parameters after exposure to the vehicle.

**Table 3:** Distributions of baseline values of the contractility parameters in 189 human ventricular cardiomyocytes from 11 donor hearts.

**Parameter**	**Mean ± s.e.m**.	**Minimum**	**Maximum**	**Median**	**Quartile 1**	**Quartile 3**
Sarcomere length (μm)	1.78 ± 0.01	1.49	1.96	1.79	1.73	1.84
Cont. vel. (μm/s)	−0.87 ± 0.02	−2.21	−0.29	−0.80	−1.07	−0.60
Sarc. short. (%)	4.31 ± 0.13	1.82	12.4	3.79	3.04	5.18
Rel. Vel. (μm/s)	0.98 ± 0.04	0.26	2.99	0.82	0.57	1.27
Peak (μm)	1.70 ± 0.01	1.21	1.89	1.72	1.64	1.78
TPeak (ms)	168 ± 3	101	341	162	140	189
TR70 (ms)	263 ± 5	147	560	251	216	308
TR80 (ms)	286 ± 6	160	620	266	229	334
TR90 (ms)	337 ± 8	189	693	314	263	403

*Cont. vel., Contraction velocity; Sarc. short., Sarcomere shortening; Ret. Vel., Relaxation velocity; Tpeak, Time to peak; TR70, TR80, and TR90, Time to 70, 80, and 90% relaxation, respectively*.

**Figure 1:**
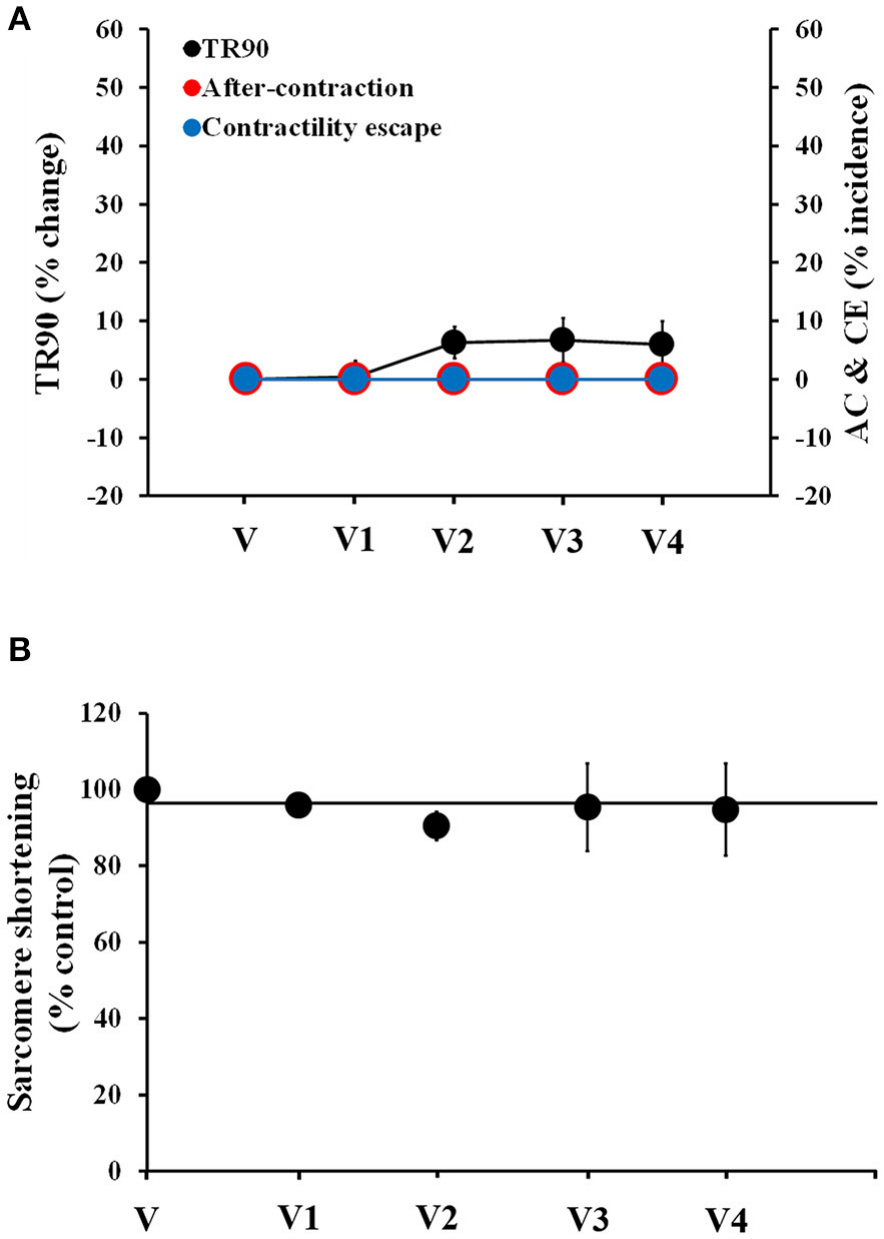
Stability of contractility recordings over time in human cardiomyocytes. **(A)** Change in TR90 and % incidence of AC and CE induced by sequential additions of vehicle (V) in human cardiomyocytes at 1 Hz pacing frequency. *P* > 0.05 vs. V-values. **(B)** Vehicle effect curve for sarcomere shortening. V1, V2, V3, and V4 correspond to the 1st, 2nd, 3rd, and 4th applications of vehicle.

Next, we assessed the stability of the human cardiomyocyte preparation. We recorded vehicle time-control data in four cardiomyocytes (one heart) using multiple additions of vehicle solution spaced by 4 min each, to mimic the experimental conditions that we were set to use with the test drugs. Cardiomyocytes exhibited stable behavior for the duration of the recordings, up to 20 min (Figure 1). No AC or CE were observed and only a small, non-significant increase in TR90 was observed (1st, 2nd, 3rd, and 4th vehicle applications increased TR90 by 0.4 ± 2, 6 ± 2, 6 ± 3, and 5 ± 4%, respectively; *p* > 0.05; Figure 1A). Similarly, the measurements of sarcomere shortening in myocytes demonstrated good stability (Figure 1B).

### Effects of torsadogenic drugs on adult human primary cardiomyocytes

**Figure 2:**
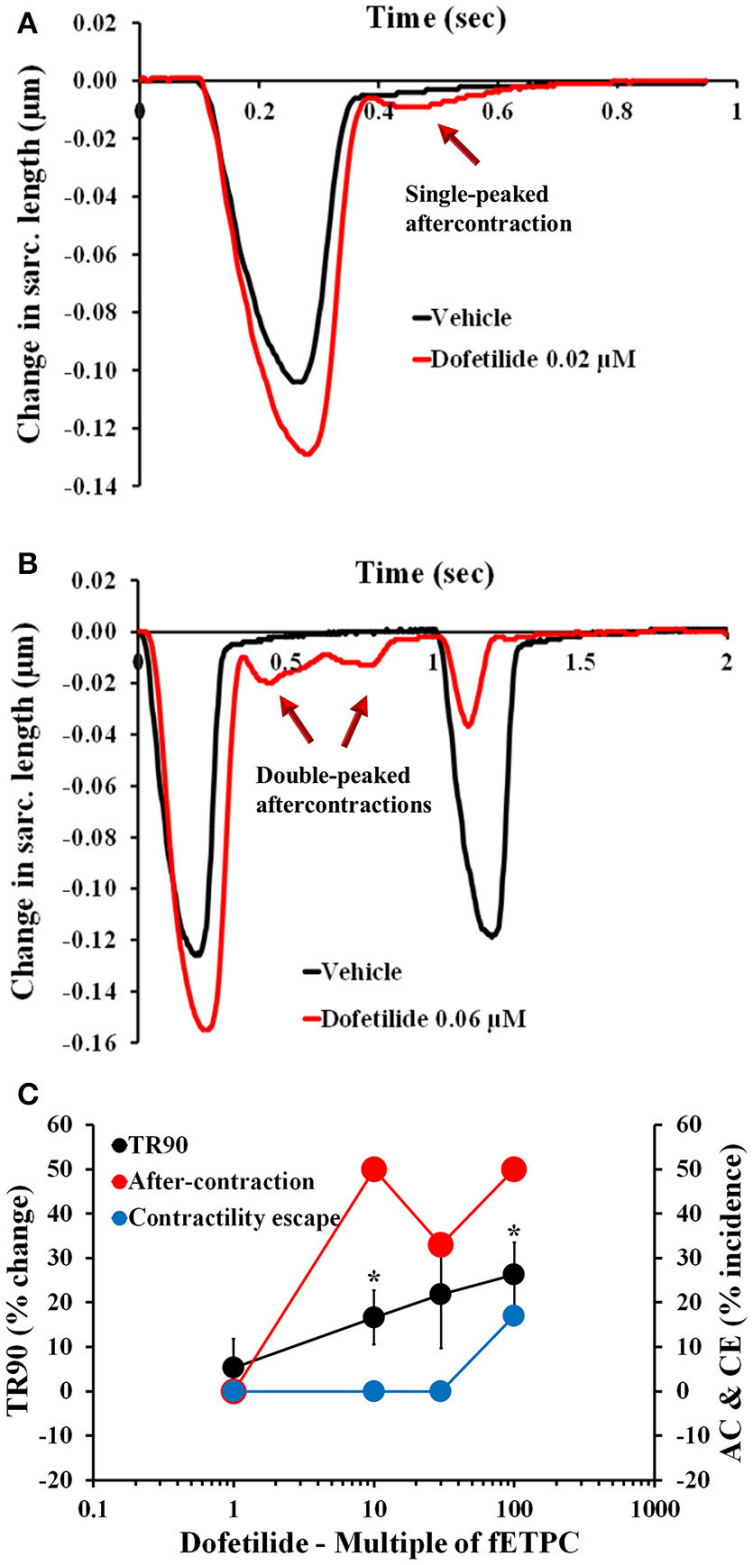
Typical contractility transients recorded from an adult human primary ventricular myocyte in the presence of vehicle control and after exposure to dofetilide at 0.02 μM **(A)**, 10-fold the fETPC, non-fitted averaged transients) and 0.06 μM (**B**, 30-fold the fETPC, non-fitted and non-averaged transients) at a pacing frequency of 1 Hz. Note that contractility transients shown in this figure were obtained from the same cardiomyocyte. **(C)** Mean % change in TR90 and AC & CE incidence when cardiomyocytes were incubated with dofetilide at 1 Hz. ^*^P < 0.05 vs. values from vehicle.

**Figure 3:**
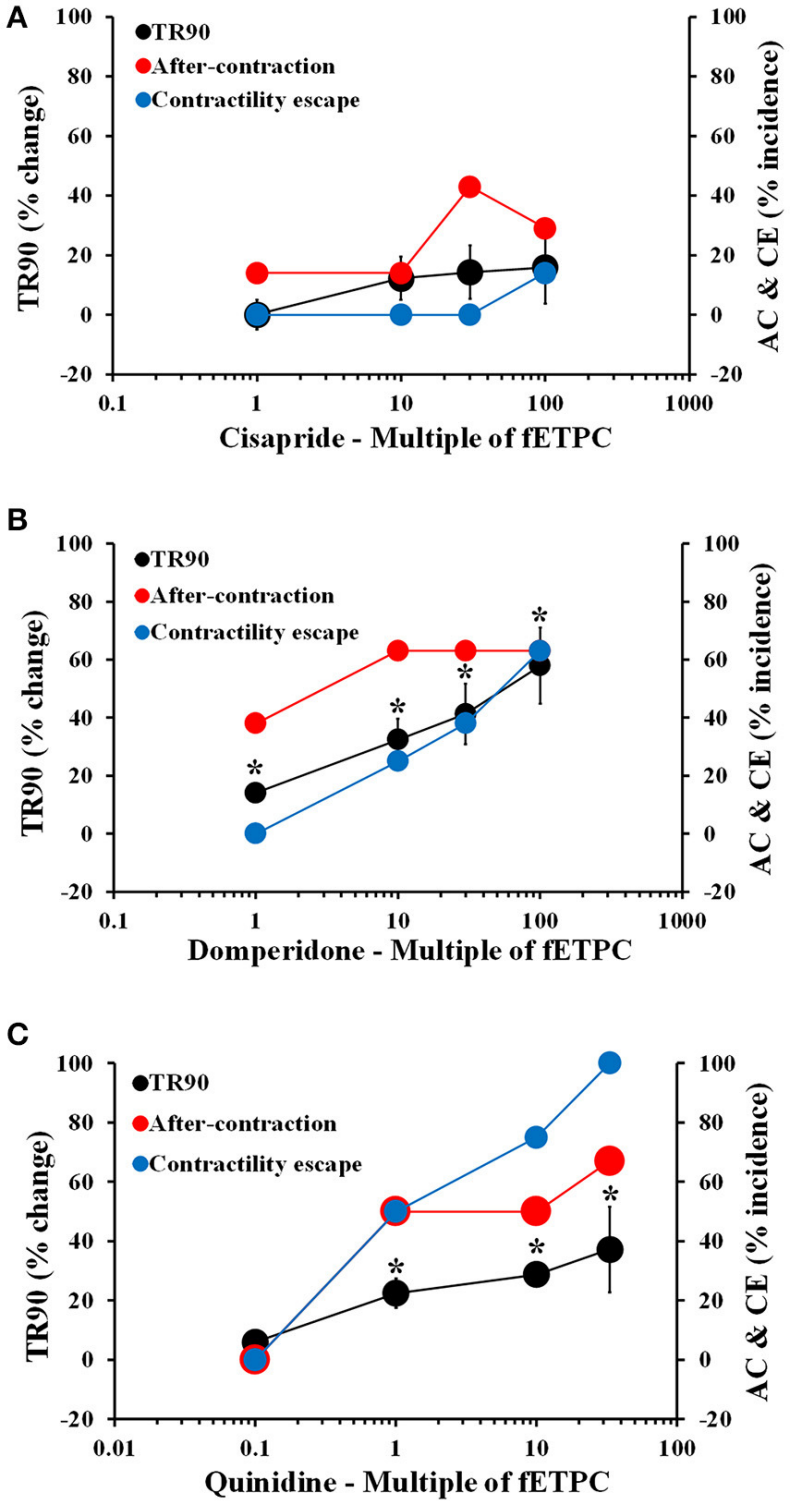
Mean % change in TR90 and AC & CE % incidence when cardiomyocytes were treated with cisapride **(A)**, domperidone **(B)** and quinidine **(C)**. ^*^P < 0.05 vs. values from vehicle.

**Figure 4:**
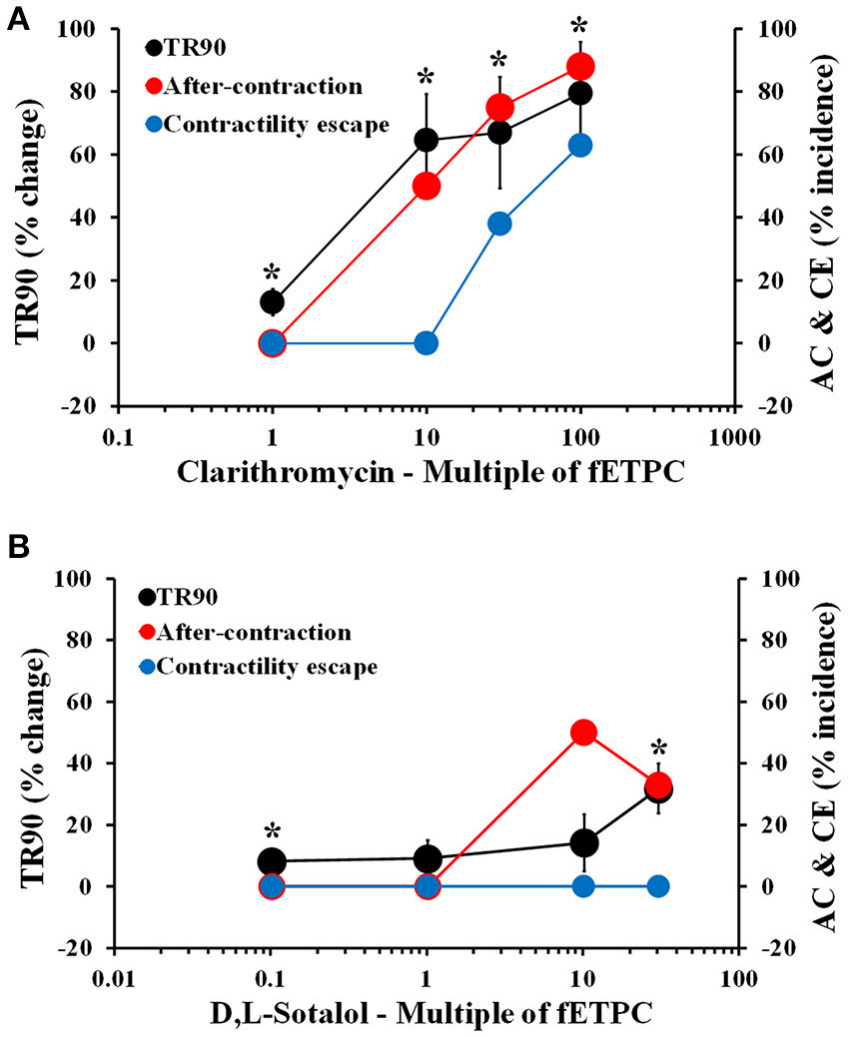
Mean % change in TR90 and AC & CE % incidence when cardiomyocytes were treated with clarithromycin **(A)** and D,L-sotalol **(B)**. ^*^P < 0.05 vs. values from vehicle.

To begin assessing the pharmacological responses of isolated adult cardiomyocytes, we selected 33 drugs, including 23 known torsadogenic (like cisapride, clarithromycin, d,l-sotalol, dofetilide, domperidone, quinidine) and 10 not previously associated with TdP arrhythmias (like mexiletine, ranolazine, verapamil; Johannesen et al., 2014; Colatsky et al., 2016; Fermini et al., 2016). Specifically, we were interested in establishing the correlation, if any, between the parameters measured in contractility transients, the clinical incidence of pro-arrhythmia and inotropic liability. The effects of the 23 torsadogenic drugs on adult human primary ventricular cardiomyocytes are shown in Figures 2–**4**, Table 4, and Supplementary Figures 4–12. Dofetilide most notably caused frequent occurrence of AC [in up to 50% of the recorded contraction transients; Figure 2C; *n* = 6 cells (1 heart)]. At the lower concentrations, the AC events consisted of a single ectopic small AC (Figure 2A), but at higher concentrations larger amplitude double-peak AC were also observed (Figure 2B). In addition, dofetilide resulted in a significant prolongation of the relaxation phase, with TR90 increase to 17 ± 6% at 10-fold of the fETPC and to 26 ± 7% at 100-fold of fETPC (Figure 2C). CE events were observed in 17% of the recordings, at the highest concentration tested (Figure 2C).

**Table 4:** Pro-arrhythmia prediction of the adult human primary cardiomyocyte-based model.

**Drug name**	**Clinical TdP risk**	**Pro-arrhythmia risk at 10-fold fETPC**					
		**AnaBios Adult human primary ventricular cardiomyocytes (Sarc. short., AC)**	**Amgen hiPSC-CMs (iCell®, MEA FPD), Qu and Vargas (2015)**	**Amgen hiPSC-CMs (iCell®, MEA EAD), Qu and Vargas (2015)**	**JiCSA hiPSC-CMs (iCell®, MEA Score), Ando et al. (2017)**	**FDA hiPSC-CMs (iCell®, MEA Arrhythmia), Blinova et al. (2017)**	**FDA hiPSC-CMs (Cor.4U, MEA Arrhythmia), Blinova et al. (2017)**
Ajmaline			Not tested	Not tested		Not tested	Not tested
Astemizole^a^		False negative	Not tested	Not tested		Not tested	Not tested
Azimilide^a^			Not tested	Not tested	Not tested	Not tested	Not tested
Bepridil^a^			Not tested	Not tested	False negative	False negative	False negative
Chlorpromazine^a^			Not tested	Not tested	False negative	False negative	False negative
Cisapride^a^				False negative		False negative	False negative
Clarithromycin^a^			Not tested	Not tested		Not tested	Not tested
Clozapine^a^			Not tested	Not tested	False negative	Not tested	Not tested
D,L-Sotalol^a^						Not tested	Not tested
Disopyramide^a^			Not tested	Not tested		Not tested	Not tested
Dofetilide^a^							
Domperidone^a^			Not tested	Not tested		Not tested	Not tested
Droperidol^a^			Not tested	Not tested		Not tested	Not tested
Erythromycin			Not tested	Not tested		Not tested	Not tested
Flecainide						Not tested	Not tested
Ibutilide^a^			Not tested	Not tested		Not tested	Not tested
Moxifloxacin				Not tested		False negative	False negative
Ondansetron^a^			Not tested	Not tested		Not tested	Not tested
Procainamide			Not tested	Not tested		Not tested	Not tested
Quinidine^a^			Not tested	Not tested			
Sematilide			Not tested	Not tested		Not tested	Not tested
Terodiline			False negative	False negative		Not tested	Not tested
Vandetanib^a^			Not tested	Not tested		Not tested	Not tested
Diltiazem^a^			Not tested	Not tested			
Diphenhydramine			Not tested	Not tested	False positive	Not tested	Not tested
Loratidine^a^			Not tested	Not tested		Not tested	Not tested
Mexiletine^a^			False positive	Not tested	False positive	Quiescent	
Mibefradil			Not tested	Not tested			
Nifedipine^a^			Not tested	Not tested		Not tested	Not tested
Nitrendipine^a^			Not tested	Not tested		Not tested	Not tested
Ranolazine^a^			False positive		False positive	False negative	
Tamoxifen^a^			Not tested	Not tested		Not tested	Not tested
Verapamil^a^			Not tested	Not tested			Quiescent

a *CiPA-selected drug; Red, Positive pro-arrhythmia risk; Green, Negative pro-arrhythmia risk; Sarc. short., Sarcomere shortening; hiPSC-CM, human induced pluripotent stem cell-derived cardiomyocyte; iCell®, hiPSC-CMs from Cellular Dynamics; MEA, Micro-electrode array; FPD, Field Potential Duration; JiCSA, Japan iPS Cardiac Safety Assessment; FDA, Food and Drug Administration; Cor.4U, hiPSC-CMs from Axiogenesis AG; EAD, Early afterdepolarization; fETPC, free effective therapeutic plasma concentration*.

Cisapride resulted in AC events at all concentrations tested and with the highest incidence at the two highest concentrations tested: 43% at 30-fold fETPC and 30% at 100-fold fETPC [Figure 3A, *n* = 7 cells (2 hearts)]. CE events were observed at the highest concentration tested in 14% of the contraction transients. No significant changes in TR90 were observed at any concentration (Figure 3A). Domperidone induced AC events at all concentrations tested, CE at all concentrations above the fETPC and significant and concentration-dependent prolongation of the relaxation phase [Figure 3B, *n* = 8 cells (1 heart)]. Quinidine and clarithromycin induced concentration-dependent increases in AC incidence, CE and prolongation of the relaxation phase [Figure 3C, *n* = 4 cells (1 heart) for quinidine; Figure 4A, *n* = 8 cells (2 hearts) for clarithromycin]. d,l-sotalol [Figure 4B, *n* = 8 cells (2 hearts)] also caused a concentration-dependent increase in TR90 and AC incidence but it did not induce CE events.

The translation predictivity of the AC parameter was used to calculate assay performance values for the adult human primary cardiomyocyte-based model (Figures 2–4; Table 4; Supplementary Figures 4–12). In comparison with clinical torsadogenic risk and when predicting pro-arrhythmic risk at 10-fold the fETPC of the 23 torsadogenic drugs, the human cardiomyocyte assay has an excellent sensitivity (96%) for predicting clinical pro-arrhythmic risk with very low false negative rate. This outstanding predictivity confirms the translational safety potential of the AC marker and sensitivity of human primary adult cardiomyocytes to the effects of the 23 torsadogenic drugs we tested; in particular this cellular preparation exhibits changes in contractility parameters that are related to the AP changes expected to be induced by the drugs (Redfern et al., 2003; CredibleMeds®, https://crediblemeds.org/). It is also important to note that the observed changes occurred at concentration ranges that are clinically relevant: all 23 drug induced contractility abnormalities, that are potentially related to pro-arrhythmia risk, starting at the fETPC.

To determine the reproducibility and reliability of adult human primary cardiomyocytes, dofetilide was tested in three donor hearts. Data summaries for the effects of dofetilide on sarcomere shortening, TR90, AC, and CE incidence are shown in Supplementary Figures 13, 14. An unmarked level of variability was seen with sarcomere shortening (Supplementary Figure 13), TR90 (Supplementary Figure 14A), AC events (Supplementary Figure 14B), and CE incidence (Supplementary Figure 14C). For example, the mean dofetilide-induced % changes in TR90 at 30-fold the fETPC were found to be 21 ± 12, 24 ± 5, and 18 ± 5% in donor hearts 1 (*n* = 6 cells), 2 (*n* = 4 cells), and 3 (*n* = 5 cells), respectively. We further assessed the level of variability by assessing the intra-heart and inter-heart (Total) variability of cell responses to dofetilide (Supplementary Figure 15). Our data show that the intra-heart variability for TR90 accounted for 90% of the Total observed variability of the TR90 parameter after exposure to dofetilide concentrations (Supplementary Figure 15A). For the inter-heart variability for the dofetilide concentration period corresponding to the top test concentration, the total SD related to the mean percent change in TR90 effects was 13.3, while the intra-heart SD for the same concentration period was 12.7. The same was true for the variability of sarcomere shortening (Supplementary Figure 15B). Taken together, these data establish that the inter-donor variability is relatively small and does not add significant noise beyond what is inherent to this experimental approach.

We also confirmed that similar data could be obtained when the experiments were conducted in blinded or non-blinded fashion. For example, the effects of ibutilide were found to be similar in blinded experiments and in unblinded testing [Supplementary Figures 16, 17; *n* = 5 blinded cells (1 heart)].

Given that canine *in-vivo* models are extensively used for drug cardiac safety assessment (Pollard et al., 2010) and isolated adult cardiomyocytes from dog hearts are also commonly tested for early risk assessment (Abi-Gerges et al., 2010; Harmer et al., 2012), we compared the effects of quinidine in human and dog adult cardiomyocytes. Quinidine elicited a significantly larger increase in TR90 in myocytes from human hearts compared to canine hearts [Supplementary Figure 18A, *n* = 5 (1 heart)]. Furthermore, AC and CE events were only observed in quinidine-treated human myocytes (Supplementary Figure 18A). These data underscore the potential limitations of canine cardiomyocyte model in recapitulating the pharmacology observed in human cardiomyocytes.

### Effects of non-torsadogenic drugs on adult human primary cardiomyocytes

**Figure 5:**
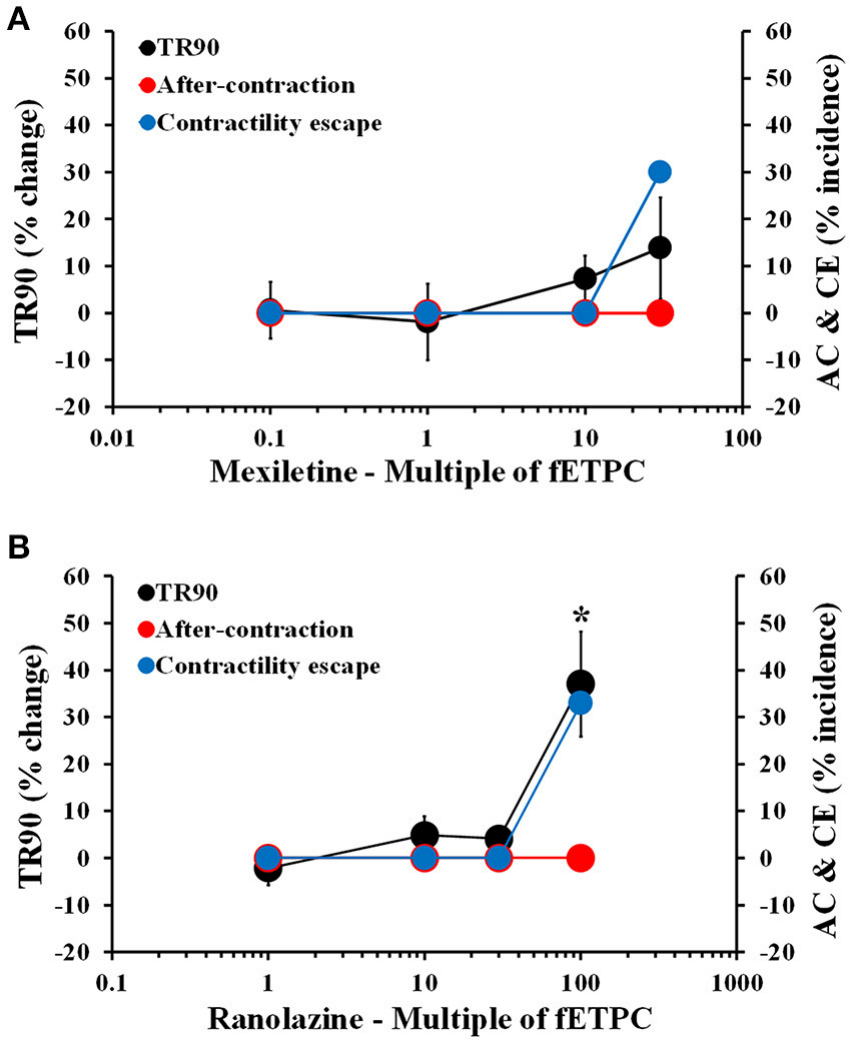
Mean % change in TR90 and AC & CE % incidence when cardiomyocytes were treated with mexiletine **(A)** and ranolazine **(B)**. ^*^P < 0.05 vs. values from vehicle.

**Figure 6:**
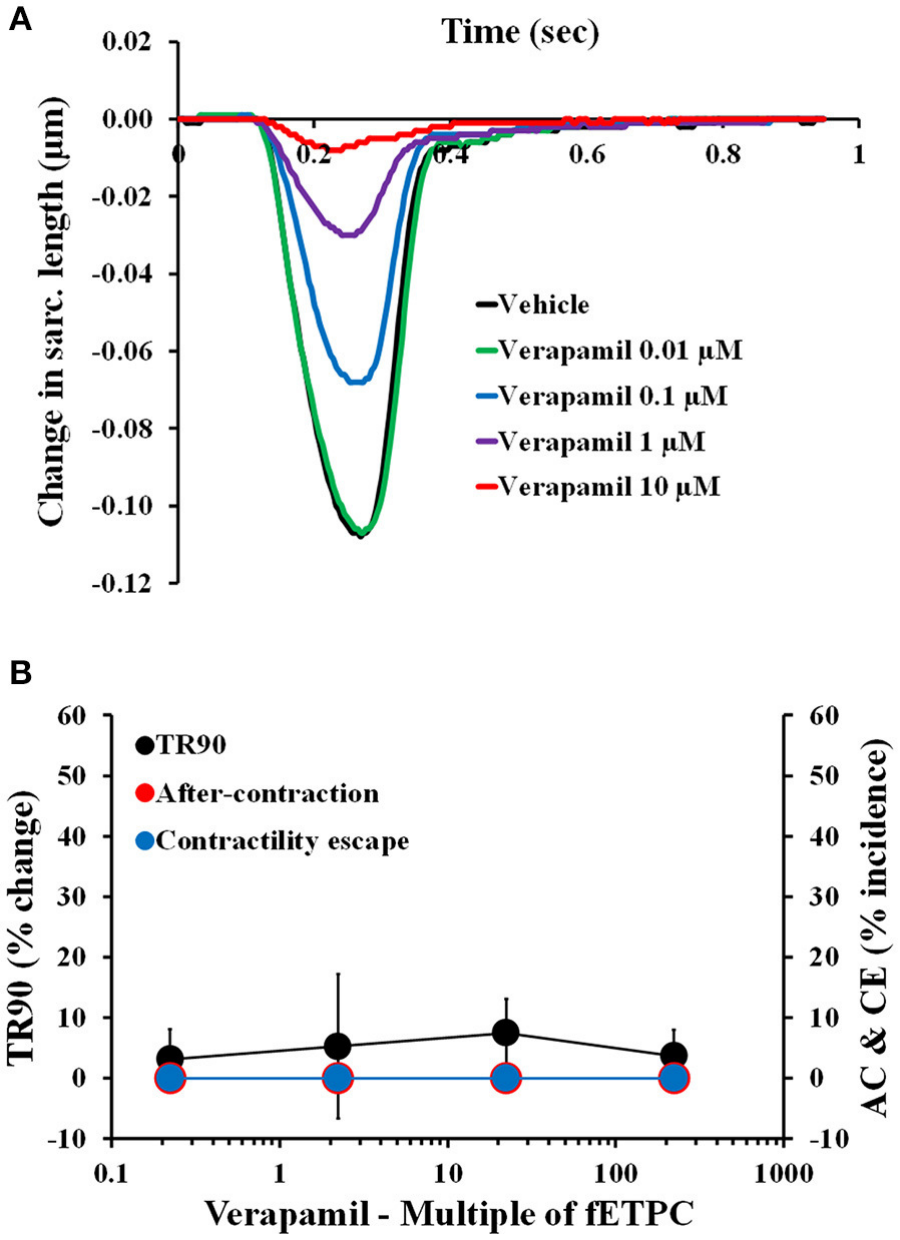
**(A)** Typical non-fitted averaged contractility transients recorded from an adult human primary ventricular myocyte in the presence of vehicle control and after exposure to verapamil at 0.01, 0.1, 1, and 10 μM (0.2-, 2-, 22-, and 222-fold the fETPC, respectively) at a pacing frequency of 1 Hz. **(B)** Mean % change in TR90 and AC & CE % incidence when cardiomyocytes were incubated with verapamil at 1 Hz. *P* > 0.05 vs. values from vehicle.

Non-torsadogenic drugs, like mexiletine, ranolazine, and verapamil, are approved drugs with low clinical torsadogenic risk (Redfern et al., 2003; Colatsky et al., 2016; Fermini et al., 2016; CredibleMeds®). While mexiletine and verapamil are not expected to delay ventricular repolarization, ranolazine can elicit prolongation of the QT interval in the electrocardiogram (ECG) (Duff et al., 1987; Giardina and Wechsler, 1990; Johannesen et al., 2014). None of the three drugs induced AC at any of the concentrations tested (Figures 5, 6). However, mexiletine induced CE events in 30% of the transients, at the highest concentration tested [30-fold the fETPC; *n* = 7 cells (1 heart); Figure 5A]. This observation is consistent with the known sodium channel inhibitory activity of mexiletine (Qu et al., 2013). Relaxation time was significantly prolonged only by ranolazine at the highest concentration tested [100-fold of fETPC; 37 ± 11%; *n* = 3 cells (1 heart); Figure 5B]; this finding is consistent with the fact that ranolazine is known to induce QT interval prolongation at concentrations above the therapeutic dose (Chaitman, 2004; Johannesen et al., 2014). Ranolazine was also able to induce CE events, which is consistent with its known inhibitory action on sodium and calcium voltage gated channels (Antzelevitch et al., 2004). The data shows that the cardiac safety margins are different for the three with mexiletine inducing CE events 10-fold above the fETPC, ranolazine above 30-fold the fETPC and verapamil not exhibiting any signal potentially predictive of pro-arrhythmia up to the highest concentration tested [220-fold of the fETPC; *n* = 4 (1 heart); Figure 6]. However, when the effects of verapamil were compared in dog and human adult cardiomyocytes, we observed that in dog cardiomyocytes verapamil induced a significant prolongation of the relaxation time: at 30- and 220-fold of fETPC, verapamil increased TR90 by 85 ± 19% [*n* = 4 cells (1 heart); Supplementary Figure 18B] and 3 ± 4% (Figure 6B), respectively. These results highlight the inability of the dog cardiomyocyte model to accurately predict the effects of verapamil on the human heart.

The AC parameter was again used to calculate specificity value for the adult human primary cardiomyocyte-based model (Figures 5, 6; Table 4; Supplementary Figures 19–21). In comparison with clinical torsadogenic risk and when predicting risk at 10-fold the fETPC of the 10 non-torsadogenic drugs, the human cardiomyocyte assay has an excellent specificity (100%) for predicting the safety of the 10 non-torsadogenic drugs. Thus, adult human primary cardiomyocytes have a great value as a specific assay to predict the safety of drugs.

### Effects of reference drugs on sarcomere shortening in adult human primary cardiomyocytes

**Figure 7:**
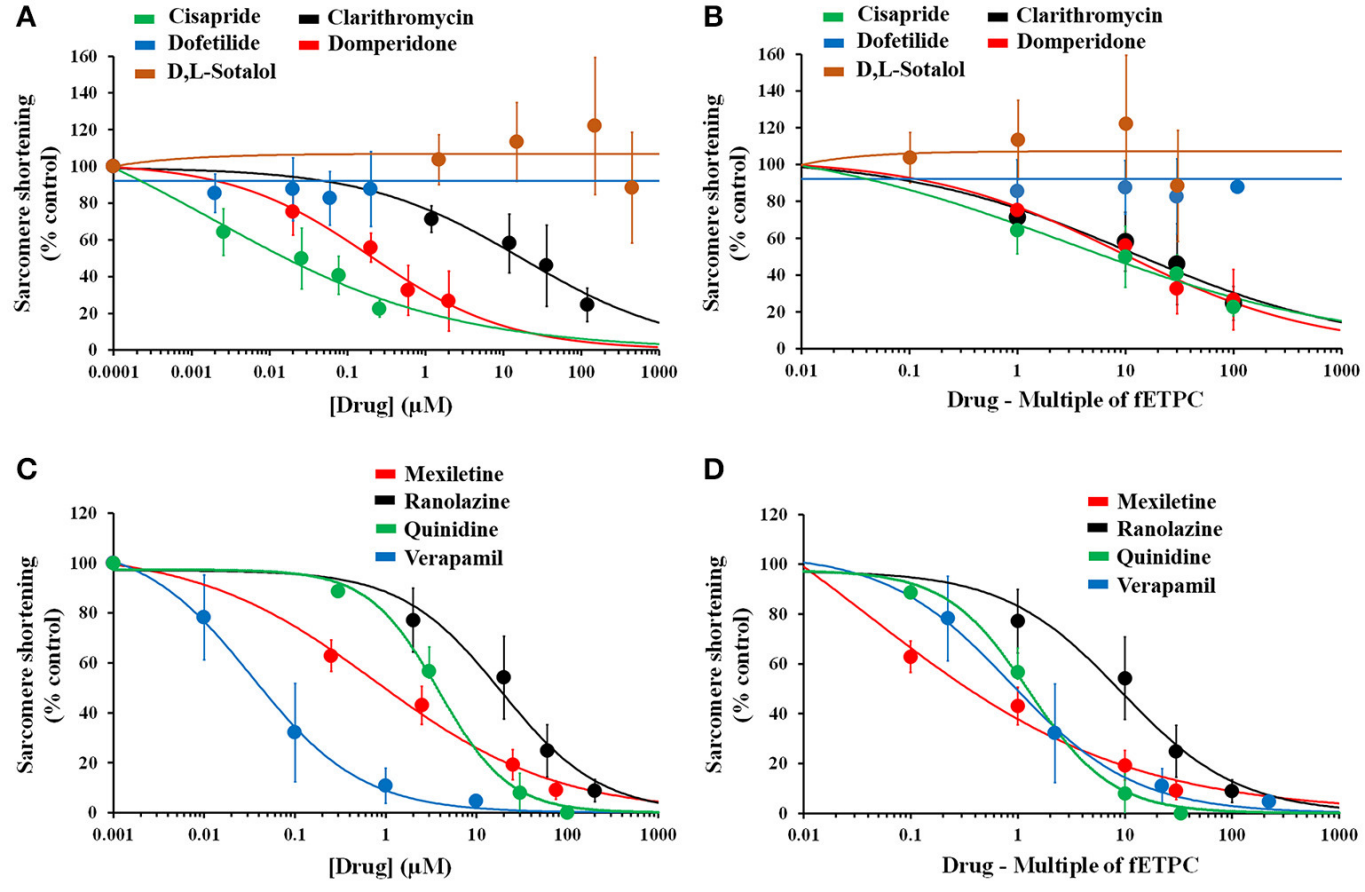
Effects of positive and negative controls on human cardiomyocyte contractility. Drug-effect curves for sarcomere shortening are shown as a function of concentrations tested **(A**,**C)** or multiple of fETPCs **(B**,**D)**. The 0.0001 and 0.001 μM represent the normalized vehicle data for drugs in **(A)** and **(B)**, respectively. IC50 (μM) and ratio (IC50/fETPC) values for the effects of multi-ion channel blockers on sarcomere shortening were found to be 0.02 and 8 for cisapride, 16 and 13 for clarithromycin, 0.2 and 10 for domperidone, 0.9 and 0.4 for mexiletine, 17 and 9 for ranolazine, 3.6 and 1 for quinidine, and 0.04 and 2 for verapamil.

We then analyzed the effects of the 33 reference drugs on sarcomere shortening in adult human primary ventricular cardiomyocytes. For example, while dofetilide and d,l-sotalol, hERG channel blockers, had no effects on sarcomere shortening (Figures 7A,B), multi-ion channel blockers, like cisapride, clarithromycin, domperidone, mexiletine, ranolazine, quinidine, and verapamil all inhibited sarcomere shortening (Figure 7). Additionally, the concentration-dependence of the negative inotropic effects of these multi-ion channel blockers (Figures 7A,C) is also evaluated in the context of the fETPC (Figures 7B,D). The same was true for other hERG channel blockers (like erythromycin, moxifloxacin and sematilide) and multi-ion channel blockers (Supplementary Figures 4–12 and 19–21; Table 5). Thus, these data demonstrate that human cardiomyocytes are of great value to screen/identify drugs associated with inotropic effects, help ranking compounds for progression to next drug discovery phases and establish human safety margins (Table 5).

**Table 5:** Sarcomere shortening effects for reference drugs measured in adult human primary cardiomyocytes.

**Drug name**	**Top test concentration (μM)**	**Human myocyte effect**	**IC_50_ (μM)**	**Ratio (IC_50_/fETPC)**
Ajmaline	1.95	−ve inotrope	2	31
Astemizole^a^	0.009	No effect	>0.009	30
Azimilide^a^	2.1	−ve inotrope	1.07	15
Bepridil^a^	0.96	−ve inotrope	0.7	22
Chlorpromazine^a^	1.04	−ve inotrope	1.02	28
Cisapride^a^	0.26	−ve inotrope	0.02	8
Clarithromycin^a^	120	−ve inotrope	16	13
Clozapine^a^	2.13	−ve inotrope	1.5	21
D, L-Sotalol^a^	450	No effect	>450	>30
Disopyramide^a^	21	−ve inotrope	9.3	13
Dofetilide^a^	0.2	No effect	>0.2	>100
Domperidone^a^	2	−ve inotrope	0.2	10
Droperidol^a^	0.48	−ve inotrope	0.18	11
Erythromycin	5.1	No effect	>5.1	>30
Flecainide	22.6	−ve inotrope	1.1	2
Ibutilide^a^	3	−ve inotrope	2	20
Moxifloxacin	329	No effect	>329	>30
Ondansetron^a^	11.2	−ve inotrope	14	34
Procainamide	1625	−ve inotrope	2215	38
Quinidine^a^	100	−ve inotrope	3.6	1
Sematilide	133	No effect	>133	>30
Terodiline	4.35	−ve inotrope	0.7	5
Vandetanib^a^	9	−ve inotrope	2.7	9
Diltiazem^a^	3.84	−ve inotrope	1	8
Diphenhydramine	1.02	−ve inotrope	0.6	17
Loratadine^a^	0.0135	−ve inotrope	0.0175	35
Mexiletine^a^	75	−ve inotrope	0.9	0.4
Mibefradil	0.36	−ve inotrope	0.18	13
Nifedipine^a^	0.23	−ve inotrope	0.04	5
Nitrendipine^a^	0.091	−ve inotrope	0.06	18
Ranolazine^a^	200	−ve inotrope	17	9
Tamoxifen^a^	0.663	−ve inotrope	0.99	36
Verapamil^a^	10	−ve inotrope	0.04	2

*IC_50_; Concentration inducing 50% decrease in sarcomere shortening; Hill equation using SigmaPlot v13 was fitted to sarcomere shortening concentration-effect curves, assuming drugs would eventually cause complete inhibition of the contractility when they decreased sarcomere shortening by ≥25%*.

a *CiPA-selected drug; fETPC, free effective therapeutic plasma concentration*.

When the effects of quinidine on sarcomere shortening were compared in human and dog cardiomyocytes, we found that the drug was 11-fold more potent in human ventricular myocytes compared to canine cells (Supplementary Figures 22A,B). Conversely, the negative inotropic effect of verapamil was similar between human and canine cells (Supplementary Figures 22C,D). These data clearly show the ability of isolated human cardiomyocytes to identify multi-ion channel drugs associated with inotropic risk and further stress the challenges in cross-species translation for cardiac risk assessment.

## Discussion

In the present work, we wanted to evaluate the potential of a novel strategy for addressing pre-clinical cardiac risk assessment. The goal was to establish and validate, a novel approach that would be: (i) human-relevant and cell-based; (ii) amenable to high-throughput screening; (iii) reliant on non-invasive measurements; (iv) simple to implement and yet able to provide a rich data set that could address both pro-arrhythmia as well as inotropic risks. We have recently established methods that enable standardized organ procurement protocols and the experimental utilization of ventricular trabeculae from human donor hearts for *ex-vivo* cardiac safety studies (Page et al., 2016). Our previous work established the low donor-to-donor variability with regards to physiological and pharmacological properties of these *ex-vivo* preparations and provided evidence for the ability of that model to distinguish between pro-arrhythmic and non-pro-arrhythmic drugs. We now further extend the previous work by reporting on the isolation and experimental interrogation of human ventricular cardiomyocytes. We describe the use of ventricular human cardiomyocytes for drug cardiac safety assessment using an *ex-vivo* model which addresses all four features discussed above: (i) the assay we developed is based on human cells; (ii) it relies on the measurement of contractility, an endpoint for which numerous options are available for performing medium- or high-throughput assays; (iii) it utilizes bright field optical imaging for measuring sarcomere shortening. This provides a non-invasive methodology which avoids the use of fluorescent dyes and the potential for chemo- or photo-toxicity; and (iv) the optically-based measurement of sarcomere shortening is simple to implement but, thanks to the utilization of refined analysis endpoints of the contractility transients, enables tracking parameters relevant to pro-arrhythmia risk as well as inotropic risks.

One critical component of our work is the utilization of data obtained from contractility measurements to infer the effects of drugs, not only with regards to inotropic effects, but also for making prediction of pro-arrhythmia risk. The justification for this approach derives from the tight functional coupling between the electrical and mechanical behavior of cardiac cells (Lou et al., 2011; Kang et al., 2016). It is well-documented that abnormal ventricular repolarization leads to contraction abnormalities: for example, delays in the repolarization phase of the cardiac AP and triggered EADs, result in delays of relaxation phase and AC events in the contraction cycle (Nador et al., 1991; De Ferrari et al., 1994; Nakayama et al., 1998; Belardinelli et al., 2009; De Ferrari and Schartz, 2009; Haugaa et al., 2009).

We first established that our methods could provide human adult myocytes exhibiting the functional parameters expected of healthy and functionally competent cardiac tissue. Our data on the contractility parameters (summarized in Table 3) are in agreement with previous reports (Gerdes et al., 1992; Davies et al., 1995, 1996; del Monte et al., 1995). Furthermore, our measurements of sarcomere shortening, as well as the findings from the previously cited papers, are all well within the range of the distance between the Z-bands (i.e., sarcomere length) of 1.6–2.2 μm in human hearts (Klabunde, 2005). Our baseline sarcomere shortening, TPeak and TR90 values agree with those reported by Lyon et al. (2009), although they are not consistent with the data reported by del Monte et al. (1995): TPeak and TR90 were higher in the del Monte study. A plausible explanation for the discrepancy is that, in del Monte study, the cardiomyocytes were paced at lower frequency, 0.2 Hz, compared to the 1 Hz pacing frequency used throughout our study. Interestingly, the TR90-values that were observed in this study and in the study by Lyon et al. (2009) are almost identical to the values previously reported for AP duration at 90% repolarization (Franz et al., 1988; Kang et al., 2016; Page et al., 2016), further supporting the functional interrelation between the electrical (AP) and mechanical (contractility) in cardiomyocytes (see also Lou et al., 2011). Additionally, cardiomyocytes obtained from 11 donor hearts showed a relatively low total variability for the contractility parameters after exposure to the vehicle control. The stability of the human adult cardiomyocyte preparation was then evaluated in time-matched vehicle control experiments. During the course of these experiments, and for the total of 20 min per experiment, no significant change was observed in sarcomere shortening and TR90, and AC or CE were not observed.

Next we assessed the effects of reference drugs with well-characterized clinical outcomes, including 23 torsadogenic and 10 non-torsadogenic drugs. Torsadogenic drugs, like dofetilide and d,l-sotalol, two hERG blockers, caused an increase of TR90 and evoked AC events starting at 10-fold fETPCs. These findings agree with clinical measurements of the QT interval following administration of these drugs, as well as reports of TdP arrhythmia for the same molecules (see, for example, Soyka et al., 1990; Torp-Pedersen et al., 1999; Johannesen et al., 2014; Colatsky et al., 2016). Moreover, dofetilide and d,l-sotalol did not significantly affect sarcomere shortening up to the highest multiple of fETPCs (100- and 30-fold, respectively). Dofetilide and d,l-sotalol lack of effect on cardiomyocyte contractility is in agreement with myocardial contractility data reported in clinical studies (FDA labels for both drugs; Brooks et al., 1970; Rasmussen et al., 1992; Holubarsch et al., 1995). Similarly to dofetilide and d,l-sotalol, other torsadogenic drugs (like cisapride, clarithromycin, domperidone, and quinidine) also increased TR90 and induced ACs. While cisapride, domperidone and quinidine induced ACs starting at fETPCs, clarithromycin induced ACs starting at 10-fold the fETPC. These findings agree with the data reported for these 4 drugs in humans (see, for example, Koster and Wellens, 1976; Roden et al., 1986; Lee et al., 1998; Vitola et al., 1998; Kamochi et al., 1999; Barbey et al., 2002; Johannes et al., 2010; van Noord et al., 2010; Johannesen et al., 2014; Colatsky et al., 2016). In contrast to dofetilide and d,l-sotalol, cisapride, clarithromycin, domperidone, and quinidine inhibited sarcomere shortening in cardiomyocytes, as had been previously shown in human myocardium (Nawrath and Eckel, 1979; Kirch et al., 1992). This effect on sarcomere shortening is in line with the ability of these drugs to simultaneously block, not only the hERG potassium channel (Redfern et al., 2003), but also other cardiac ion channels, like Na^+^ and Ca^2+^ channels (Gluais et al., 2003; Harmer et al., 2011; Mirams et al., 2011; Kramer et al., 2013; Crumb et al., 2016). The remaining 17 torsadogenic drugs displayed similar torsadogenic and inotropic behaviors. Additionally, AC incidence seen at fETPCs in our study is consistent with reports of TdP cases with therapeutic concentrations (like with quinidine; Koster and Wellens, 1976; Roden et al., 1986). TdP risk is also known to increase with increasing concentrations as a result of administering a high dose or drug accumulation in plasma or in cardiac tissue (Mounsey and DiMarco, 2000; Reiffel and Appel, 2001). Such a dose-risk relationship was observed in our study in which AC incidence increased as the testing concentration was elevated. Moreover, human cardiomyocytes identified with excellent sensitivity (96%) drugs associated with pro-arrhythmic risk, displayed consistent reproducibility of ibutilide- and dofetilide-induced inotropic and pro-arrhythmia risk with a relatively low total variability of the pharmacological response to dofetilide. Altogether, our data with the 23 torsadogenic drugs support the potential of these human cardiomyocytes, combined with measurement of contractility transients, to significantly enhance preclinical cardiac safety assessment by stopping true positive compounds from being developed as novel therapies. Pacing frequency may influence kinetic drug binding in ion channels and usage of one pacing frequency may lead to false negative outcomes. However, human cardiomyocytes assessed at only 1 Hz pacing frequency (our study) had an excellent sensitivity. This indicates that these 1 Hz-paced cells would only be associated with 4% chance in incorrectly categorizing drugs as false negatives. If the chemical space of a drug discovery project is found to be frequency-dependent, re-assessment of 1 Hz-categorized true negative compounds at slower or faster pacing rate would be recommended. Finally, cell-to-cell coupling may attenuate AC events in multicellular cardiac preparations compared to isolated uncoupled cardiomyocytes. Preliminary findings show that ventricular trabeculae, like human cardiomyocytes, could differentiate between the safety of ranolazine and the torsadogenic potential of dofetilide, and identify the inotropic risk associated with ranolazine (data not shown). Although these data are very encouraging, a future study is necessary to determine the influence of cell-to-cell coupling on the prediction of drug-induced pro-arrhythmic risk.

The 10 non-pro-arrhythmic drugs used in this study are multi-ion channel blockers (Liu et al., 1998; He et al., 2003; Antzelevitch et al., 2004; Kramer et al., 2013; Anon, 2014; Crumb et al., 2016); possibly due to their multi-ion channel activity, they were also able to decrease sarcomere shortening in human isolated cardiomyocytes. Importantly though, none of these non-pro-arrhythmic drugs induced AC events, even when tested at large multiples of fETPCs. For example, mexiletine, ranolazine, and verapamil induced no AC events at 30-, 100- and 222-fold above fETPCs, respectively. The lack of clinical QT interval prolongation and pro-arrhythmia risk with these three drugs (see, for example, Ritchie et al., 2006; Johannesen et al., 2014; Vicente et al., 2015) has been explained with their ability to simultaneously inhibit the hERG channel and Ca^2+^ channels (verapamil; Vicente et al., 2015; Crumb et al., 2016) or late Na^+^ inward currents (mexiletine and ranolazine; Johannesen et al., 2016; Vicente et al., 2016). In fact, these electrophysiological effects may explain the anti-arrhythmic activity of mexiletine and ranolazine (Duff et al., 1987; Giardina and Wechsler, 1990; Moss et al., 2008). In agreement with our sarcomere shortening data, verapamil and mexiletine (dosed at high multiples of the therapeutic plasma levels) were found to reduce contractility and cardiac ejection fraction (Gottlieb and Weinberg, 1992; Ritchie et al., 2006). Moreover, mexiletine (Shanks, 1984; Stein et al., 1984; Sami and Lisbona, 1985) and ranolazine (Murray and Colombo, 2014) were shown to not affect contractility at therapeutic plasma levels. This emphasizes the importance of assessing drug effects as a function of the fETPC. Therefore, use of C-E curves normalized to the fETPC enables a more accurate ranking of drug risk and consequently more educated decision at early drug discovery stage. Consequently, human cardiomyocytes identified with excellent specificity (100%) the safety of the 10 non-torsadogenic drugs tested in this study and, when combined with measurement of contractility, they may have a great value in identifying true negative compounds and hence supporting the development of new drugs without inotropic and pro-arrhythmia risk.

Side by side comparison in human and canine adult cardiomyocytes for two of the compounds highlighted the potential for interspecies differences in pharmacological responses. In our experiments cardiomyocytes from dog exhibited limited sensitivity to the effects of quinidine, with a right shift in the concentration dependence of TR90 prolongation and no observed AC or CE events, which in our model would result in underestimation of the pro-arrhythmic risk of this drug. In addition, quinidine had a more potent negative inotropic effect in human compared to dog myocytes. The underlying cause for these discrepancies could be the different affinities of the drug for canine and human K^+^, Na^+^, and Ca^2+^ channels; it is also possible that species-specific differences in the relative levels of expression of channels responsible for inward and outward currents, may lead to the discrepancy in pharmacological responses. In the case of verapamil, both human and dog myocytes exhibited similar inotropic effects, but in dog myocytes a significant prolongation of the TR90 was observed, which was not measured in the human cells. Such a lack of cross species consistency of drug effects is an obvious concern, given how much reliance is still placed on the use of animal models for complex *in-vivo* cardiovascular safety studies. Given the discrepancies that we and others have highlighted (Perel et al., 2007; Seok et al., 2013), it would seem prudent to assess each new drug candidate using the approach we have described to circumvent the translatability issues of the animal model.

Recent efforts to develop and validate new robust, reliable and predictive human cardiac safety assessment tools (Sager et al., 2014; Holmes et al., 2015; Gintant et al., 2017) have been focused primarily on human stem cell-derived cardiomyocytes (hSC-CMs) (see, for example, Zhao et al., 2016; Gintant et al., 2017). It has been pointed out that hSC-CM lack several features found in their adult primary homologs (van Meer et al., 2016) and attempts at improving the extend of hSC-CMs maturation have been made (Veerman et al., 2015; Sala et al., 2016). In Table 4 we have summarized the findings of different studies in which the same 33 drugs presented in this study were used. While the degree of success of hSC-CMs in correctly classifying pro- and non-proarrhythmic drugs varies, it is also apparent that hSC-CMs have a particularly high rate of false positive and false negative findings when multi-ion channel blockers are tested. This is not surprising, given the known challenges in fully differentiating these cells into a desired cardiac subtype and maturing them to the adult phenotype, which most likely results in non-physiological levels of expression of the conductances that govern the cardiac AP (Qu et al., 2013; Blinova et al., 2017).

Another major initiative currently underway to improve the existing cardiac safety paradigm is the CiPA (Comprehensive *in vitro* Pro-arrhythmia Assay; Sager et al., 2014; Fermini et al., 2016). Functional assessment of drug effects on multiple cardiac ion channels from cell lines and *in-silico* modeling of drug effects, to generate a pro-arrhythmia score, are the core elements of the CiPA initiative (Sager et al., 2014). Under the strategy being currently evaluated, CiPA-derived prediction of risk could then be confirmed in hSC-CMs (Sager et al., 2014; Colatsky et al., 2016; Crumb et al., 2016; Fermini et al., 2016; Gintant et al., 2016; Li et al., 2017; Windley et al., 2017). Each element of the CiPA strategy faces significant challenges. Predictive *in-silico* modeling of drug effects critically depends on the accurate measurement of drug effects for each one of the ion channels included in the simulation (Fermini et al., 2016). This is of fundamental importance both at the stage of algorithm parameters' tuning as well as at the later stage of drug risk evaluation. While the experimental measurement of IC_50_ for each one of the channels being modeled is a seemingly straightforward task, two often overlooked challenges, undermine the reliability of these measurements. While many technologies are available for obtaining precise measurements of the concentration-response inhibition curves, obtaining accurate measurements is extremely difficult. In particular, a very large proportion of small molecules active on the principal inward conductances (Na^+^ and Ca^2+^ inhibitors) exhibit use dependence. This renders the magnitude of observed inhibition completely dependent upon both the specific voltage waveform as well as the stimulation frequency. Therefore, a truly accurate IC_50_ could only be obtained by performing the measurement using voltage clamp recordings while stimulating the cells with the cardiac AP waveform at the physiological rate of about 1 Hz. Technical and biological constraints render this experimental design extremely challenging and impractical with the result that the IC_50_ for the inward conductances are often not accurate. This is compounded by the second challenge, which is created by the fact that the cardiac AP is the result of the non-linear interaction of many inward and outward conductances. The non-linearity amplifies the effects of errors in the IC_50_, when one attempts to combine all the drug's effect on the various ion channels in a simulation aimed at modeling the pharmacological effects on the cardiac AP.

In principle, human adult primary cardiomyocytes could bypass all the above-mentioned challenges and limitations. These cells provide a naturally integrated system and are the minimal unit recapitulating all the key features of cardiac function: AP generation and excitation-contraction coupling. By virtue of their derivation from human adult hearts, they do not require any re-engineering or other artificial manipulation of their gene expression profile. In fact, they could provide the most clinically relevant model for the early assessment of potential cardiac risks of new drugs. This strategy would require adequate throughput to enable the screening of tens to hundreds of molecules per week. The endpoint we have used in the present study provide both low technical complexity and high degree of information with regards to drug's effect and pro-arrhythmia and inotropic risks. Recent technological developments hold great promises for the ability to implement optically based contractility measurements in high throughput platforms and could greatly facilitate the adoption of this innovative approach. Importantly, the data generated in the model we have developed, could be used to fine tune the parameters of *in-silico* models of the human heart (see Britton et al., 2017), without requiring any reliance on difficulty to measure individual ion channel effects. The *in-silico* models could then be invaluable for deconvoluting the signals that a drug may generate in the human adult myocyte assay, providing specific guidance as to the mechanism underpinning the observed signals and therefore guiding targeted medicinal chemistry effort to remove the undesired activity. This new paradigm may potentially have the following core elements: (i) Functional evaluation of drug effects on human ventricular myocytes; (ii) modeling-based deconvolution of the observed drug effects, if any, and identification of the potential undesired activities; (iii) mitigation of the liability with medicinal chemistry; and (iv) confirmation of successful elimination of the liability in cardiomyocytes. If the compound is found not to be associated with inotropic and pro-arrhythmia risk, it could simply progress to next discovery milestone. Finally, in addition to the study of normal adult human primary cardiomyocytes presented in the present study, the opportunity now exists for the use of adult cardiomyocytes from highly prevalent disease conditions (diabetes, cardiac hypertrophy, heart failure, etc.) or disease- and patient-specific hSC-CM lines, and therefore, for the ability to assess how cardiac toxicity risk may be affected by common comorbidities.

In conclusion, the results of the present investigation suggest that the adult human primary cardiomyocyte-based model has the potential to simultaneously predict risk associated with inotropic activity and pro-arrhythmia, and enables, for the first time, the generation of reliable and predictive human cardiotoxicity data during early phases of the drug discovery process.

## Author contributions

NN, GP, PM, AG, and NA-G: designed the study; NN, WN, BN, and PR: performed experiments; NN, WN, GP, and NA-G: analyzed data; PM, AG, and NA-G: wrote the article.

### Conflict of interest statement

All authors are employed by AnaBios Corporation.
