# Supplementary figures and images for: Adult Human Primary Cardiomyocyte-Based Model for the Simultaneous Prediction of Drug-Induced Inotropic and Pro-arrhythmia Risk
Source: Front Physiol. 2017 Dec 19;8:1073. doi: 10.3389/fphys.2017.01073 (PMC5742250; doi:10.3389/fphys.2017.01073)

## Slide 1
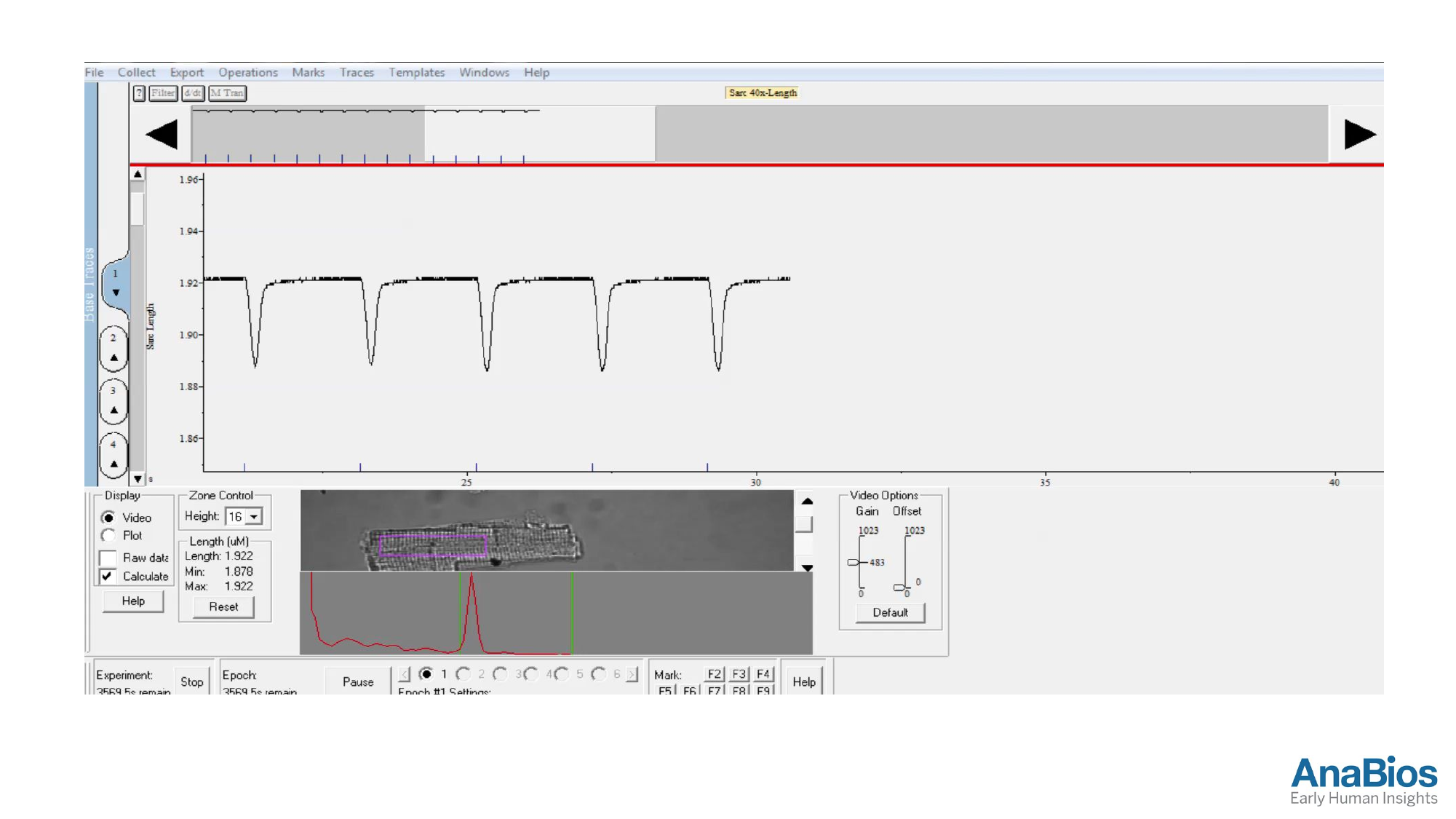

Supplement: Supplementary file 1 [file SupplementaryVideo1.PPTX]
