# Supplementary material for: Adult Human Primary Cardiomyocyte-Based Model for the Simultaneous Prediction of Drug-Induced Inotropic and Pro-arrhythmia Risk
Source: Front Physiol. 2017 Dec 19;8:1073. doi: 10.3389/fphys.2017.01073 (PMC5742250; doi:10.3389/fphys.2017.01073)
Supplement: Supplementary file 2 [file LegendsofSupplementaryFigures.DOCX]

Supplementary Material

Adult Human Primary Cardiomyocyte-based Model for the Simultaneous

Prediction of Drug-induced Inotropic and Pro-arrhythmia Risk

Nathalie Nguyen ^1^, William Nguyen ^1^, Brynna Nguyenton ^1^, Phachareeya Ratchada ^1^,

Guy Page ^1^, Paul E Miller ^1^, Andre Ghetti ^1^, Najah Abi-Gerges ^1٭^

^1^ AnaBios Corporation, San Diego, CA 92109, USA

Running title: Human cardiac safety assessment of drugs

^*^Author for correspondence:

Dr. Najah Abi-Gerges

3030 Bunker Hill St., Suite 312

San Diego, CA  92109  USA
Tel: +1 858-224-7360 (Extension 207)

Email: [Najah.abigerges@anabios.com](mailto:Najah.abigerges@anabios.com)

**SUPPLEMENTARY** **FIGURE LEGENDS**

**Supplementary Figure 1** **|** Shows a typical human heart that AnaBios uses to isolate cardiomyocytes and phase contrast microscopy images of representative adult human primary cardiomyocytes. Isolated cardiomyocytes were found to be Ca^2+^-tolerant, retain rod-shaped morphology and exhibit cross striations.

**Supplementary Figure 2** **|** Distribution histograms of sarcomere shortening, TR90 and TPeak in adult human primary cardiomyocytes at a pacing rate of 1 Hz.

**Supplementary Figure 3** **|** Variability in measurements performed from 11 donor hearts and from different adult human primary cardiomyocytes from the same heart. Figure shows the “Total” and “intra-heart” variabilities for sarcomere shortening, TPeak, TR70, TR80 and TR90 at a pacing rate of 1 Hz (n = 189). SD, Standard deviation; Sarc. short., sarcomere shortening; TPeak, Time to peak; TR70, TR80 and TR90, Time to 70%, 80% and 90% relaxation, respectively.

**Supplementary Figure 4** **|** Mean % change in TR90 and AC & CE % incidence when cardiomyocytes were treated with ajmaline **(A)** and astemizole **(C)**. P>0.05 versus TR90 values from vehicle. **(B)** and **(D)** Effects of ajmaline and astemizole on human cardiomyocyte contractility, respectively. Drug-effect curves for sarcomere shortening are shown as a function of multiple of fETPCs. The 0.1-fold represents the normalized vehicle data for both drugs in **(B)** and **(D)**. Results are expressed as mean ± SEM. fETPC, free Effective Therapeutic Plasma Concentration.

**Supplementary Figure 5** **|** Mean % change in TR90 and AC & CE % incidence when cardiomyocytes were treated with azimilide **(A)** and bepridil **(C)**. ^*^P<0.05 versus TR90 values from vehicle. **(B)** and **(D)** Effects of azimilide and bepridil on human cardiomyocyte contractility, respectively. Drug-effect curves for sarcomere shortening are shown as a function of multiple of fETPCs. The 0.1-fold represents the normalized vehicle data for both drugs in **(B)** and **(D)**. Results are expressed as mean ± SEM. fETPC, free Effective Therapeutic Plasma Concentration.

**Supplementary Figure 6** **|** Mean % change in TR90 and AC & CE % incidence when cardiomyocytes were treated with chlorpromazine **(A)** and clozapine **(C)**. ^*^P<0.05 versus TR90 values from vehicle. **(B)** and **(D)** Effects of chlorpromazine and clozapine on human cardiomyocyte contractility, respectively. Drug-effect curves for sarcomere shortening are shown as a function of multiple of fETPCs. The 0.1-fold represents the normalized vehicle data for both drugs in **(B)** and **(D)**. Results are expressed as mean ± SEM. fETPC, free Effective Therapeutic Plasma Concentration.

**Supplementary Figure 7** **|** Mean % change in TR90 and AC & CE % incidence when cardiomyocytes were treated with disopyramide **(A)** and droperidol **(C)**. ^*^P<0.05 versus TR90 values from vehicle. **(B)** and **(D)** Effects of disopyramide and droperidol on human cardiomyocyte contractility, respectively. Drug-effect curves for sarcomere shortening are shown as a function of multiple of fETPCs. The 0.1-fold represents the normalized vehicle data for both drugs in **(B)** and **(D)**. Results are expressed as mean ± SEM. fETPC, free Effective Therapeutic Plasma Concentration.

**Supplementary Figure 8** **|** Mean % change in TR90 and AC & CE % incidence when cardiomyocytes were treated with erythromycin **(A)** and flecainide **(C)**. ^*^P<0.05 versus TR90 values from vehicle. **(B)** and **(D)** Effects of erythromycin and flecainide on human cardiomyocyte contractility, respectively. Drug-effect curves for sarcomere shortening are shown as a function of multiple of fETPCs. The 0.1-fold represents the normalized vehicle data for both drugs in **(B)** and **(D)**. Results are expressed as mean ± SEM. fETPC, free Effective Therapeutic Plasma Concentration.

**Supplementary Figure 9** **|** Mean % change in TR90 and AC & CE % incidence when cardiomyocytes were treated with ibutilide **(A)** and moxifloxacin **(C)**. P>0.05 versus TR90 values from vehicle. **(B)** and **(D)** Effects of ibutilide and moxifloxacin on human cardiomyocyte contractility, respectively. Drug-effect curves for sarcomere shortening are shown as a function of multiple of fETPCs. The 0.1-fold represents the normalized vehicle data for both drugs in **(B)** and **(D)**. Results are expressed as mean ± SEM. fETPC, free Effective Therapeutic Plasma Concentration.

**Supplementary Figure 10** **|** Mean % change in TR90 and AC & CE % incidence when cardiomyocytes were treated with ondansetron **(A)** and procainamide **(C)**. P>0.05 versus TR90 values from vehicle. **(B)** and **(D)** Effects of ondansetron and procainamide on human cardiomyocyte contractility, respectively. Drug-effect curves for sarcomere shortening are shown as a function of multiple of fETPCs. The 0.1-fold represents the normalized vehicle data for both drugs in **(B)** and **(D)**. Results are expressed as mean ± SEM. fETPC, free Effective Therapeutic Plasma Concentration.

**Supplementary Figure 11** **|** Mean % change in TR90 and AC & CE % incidence when cardiomyocytes were treated with sematilide **(A)** and terodiline **(C)**. ^*^P<0.05 versus TR90 values from vehicle. **(B)** and **(D)** Effects of sematilide and terodiline on human cardiomyocyte contractility, respectively. Drug-effect curves for sarcomere shortening are shown as a function of multiple of fETPCs. The 0.1-fold represents the normalized vehicle data for both drugs in **(B)** and **(D)**. Results are expressed as mean ± SEM. fETPC, free Effective Therapeutic Plasma Concentration.

**Supplementary Figure 12** **|** Mean % change in TR90 and AC & CE % incidence when cardiomyocytes were treated with vandetanib **(A)** and diltiazem **(C)**. ^*^P<0.05 versus TR90 values from vehicle. **(B)** and **(D)** Effects of vandetanib and diltiazem on human cardiomyocyte contractility, respectively. Drug-effect curves for sarcomere shortening are shown as a function of multiple of fETPCs. The 0.1-fold represents the normalized vehicle data for both drugs in **(B)** and **(D)**. Results are expressed as mean ± SEM. fETPC, free Effective Therapeutic Plasma Concentration.

**Supplementary Figure 13** **|** Variability in dofetilide sarcomere shortening effect. Dofetilide-effect curves for sarcomere shortening are shown as a function of concentrations tested **(A)** or multiple of fETPCs **(B)** on 3 separate donor hearts. The 0.001 µM and 0.1-fold represent the normalized vehicle data for **(A)** and **(B)**, respectively. Results are expressed as mean ± SEM. fETPC, free Effective Therapeutic Plasma Concentration.

**Supplementary Figure 14** **|** Mean % change in TR90 **(A)** and AC **(**A**)** & CE **(C)** % incidence when cardiomyocytes were treated with dofetilide on 3 separate donor hearts. fETPC, free Effective Therapeutic Plasma Concentration. Results are expressed as mean ± SEM. ^*,*,*^P<0.05 versus TR90 values from vehicle. P>0.05 donor 1 TR90 values vs. donor 2 TR90 values, donor 1 TR90 values vs. donor 3 TR90 values or donor 2 TR90 values vs. donor 3 TR90 values.

**Supplementary Figure 15** **|** Variability in measurements performed from 3 donor hearts and from different adult human primary cardiomyocytes from the same heart. **(A)** and **(B)** show the “Total” and “intra-heart” variabilities for TR90 and sarcomere shortening at a pacing rate of 1 Hz, respectively. Conc., Concentration; SD, Standard deviation; Sarc. short., sarcomere shortening.

**Supplementary Figure 16** **|** Variability in ibutilide sarcomere shortening potency. Ibutilide-effect curves for sarcomere shortening are shown as a function of concentrations tested **(A)** or multiple of fETPCs **(B)** on 2 separate donor hearts. The 0.01 µM and 0.1-fold represent the normalized vehicle data for **(A)** and **(B)**, respectively. Results are expressed as mean ± SEM. fETPC, free Effective Therapeutic Plasma Concentration.

**Supplementary Figure 17** **|** Mean % change in TR90 **(A)** and AC **(**A**)** & CE **(C)** % incidence when cardiomyocytes were treated with ibutilide on 2 separate donor hearts. fETPC, free Effective Therapeutic Plasma Concentration. Results are expressed as mean ± SEM. P>0.05 versus TR90 values from unblind values.

**Supplementary Figure 18** **|** (**A** and **B**) Quinidine elicited a significantly higher increase in TR90 in myocytes from human hearts compared to canine hearts (**A**), whereas verapamil elicited a significantly higher increase in this parameter in canine compared to human myocytes (**B**). fETPC, free Effective Therapeutic Plasma Concentration; TR90, Time to 90% relaxation. Results are expressed as mean ± SEM. ^*^P<0.05 versus TR90 values from vehicle.

**Supplementary Figure 19** **|** Mean % change in TR90 and AC & CE % incidence when cardiomyocytes were treated with diphenhydramine **(A)** and loratadine **(C)**. P>0.05 versus TR90 values from vehicle. **(B)** and **(D)** Effects of diphenhydramine and loratadine on human cardiomyocyte contractility, respectively. Drug-effect curves for sarcomere shortening are shown as a function of multiple of fETPCs. The 0.1-fold represents the normalized vehicle data for both drugs in **(B)** and **(D)**. Results are expressed as mean ± SEM. fETPC, free Effective Therapeutic Plasma Concentration.

**Supplementary Figure 20** **|** Mean % change in TR90 and AC & CE % incidence when cardiomyocytes were treated with mibefradil **(A)** and nifedipine **(C)**. ^*^P<0.05 versus TR90 values from vehicle. **(B)** and **(D)** Effects of mibefradil and nifedipine on human cardiomyocyte contractility, respectively. Drug-effect curves for sarcomere shortening are shown as a function of multiple of fETPCs. The 0.1-fold represents the normalized vehicle data for both drugs in **(B)** and **(D)**. Results are expressed as mean ± SEM. fETPC, free Effective Therapeutic Plasma Concentration.

**Supplementary Figure 21** **|** Mean % change in TR90 and AC & CE % incidence when cardiomyocytes were treated with nitrendipine **(A)** and tamoxifen **(C)**. P>0.05 versus TR90 values from vehicle. **(B)** and **(D)** Effects of nitrendipine and tamoxifen on human cardiomyocyte contractility, respectively. Drug-effect curves for sarcomere shortening are shown as a function of multiple of fETPCs. The 0.1-fold represents the normalized vehicle data for both drugs in **(B)** and **(D)**. Results are expressed as mean ± SEM. fETPC, free Effective Therapeutic Plasma Concentration.

**Supplementary Figure 22** **|** Quinidine and verapamil sarcomere shortening potencies in human and dog cardiomyocytes. Quinidine- and verapamil-effect curves for sarcomere shortening are shown as a function of concentrations tested **(A** and **C**, respectively**)** or multiple of fETPCs **(B** and **D**, respectively**)** on 2 separate donor hearts. The 0.01 and 0.001µM represent the normalized vehicle data for **(A)** and **(C)**, respectively. The 0.01-fold represent the normalized vehicle data for **(B)** and **(D)**, respectively. Results are expressed as mean ± SEM. fETPC, free Effective Therapeutic Plasma Concentration; IC50, Concentration evoking a 50% decrease in sarcomere shortening; IM50, margin evoking a 50% decrease in sarcomere shortening.

**Supplementary Video 1** **|** The IonOptix platform, a video-based cell geometry system, was used to measure sarcomere dynamics from a 1Hz field-stimulated adult human primary cardiomyocyte at 0.5Hz (mimicking slow rate), 1Hz (mimicking normal rate) and 2Hz (mimicking fast rate) pacing frequencies. Note that this video shows a fundamental property of adult human primary cardiomyocytes: the ability to adapt to an increase in the pacing rate with an increase in sarcomere shortening.
