# Supplementary material for: Adult Human Primary Cardiomyocyte-Based Model for the Simultaneous Prediction of Drug-Induced Inotropic and Pro-arrhythmia Risk
Source: Front Physiol. 2017 Dec 19;8:1073. doi: 10.3389/fphys.2017.01073 (PMC5742250; doi:10.3389/fphys.2017.01073)
Supplement: Supplementary file 3 [file SupplementaryFigures.PDF]

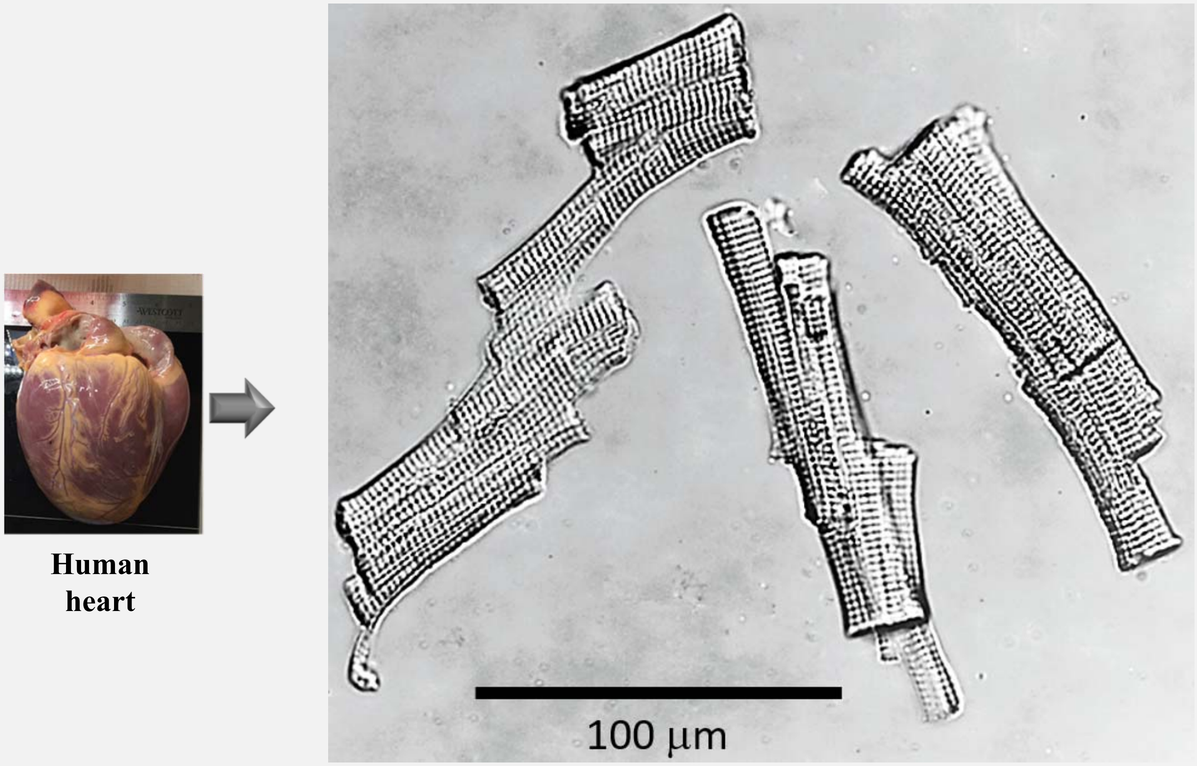

Adult Human Primary
Cardiomyocytes

Supplementary Figure 1

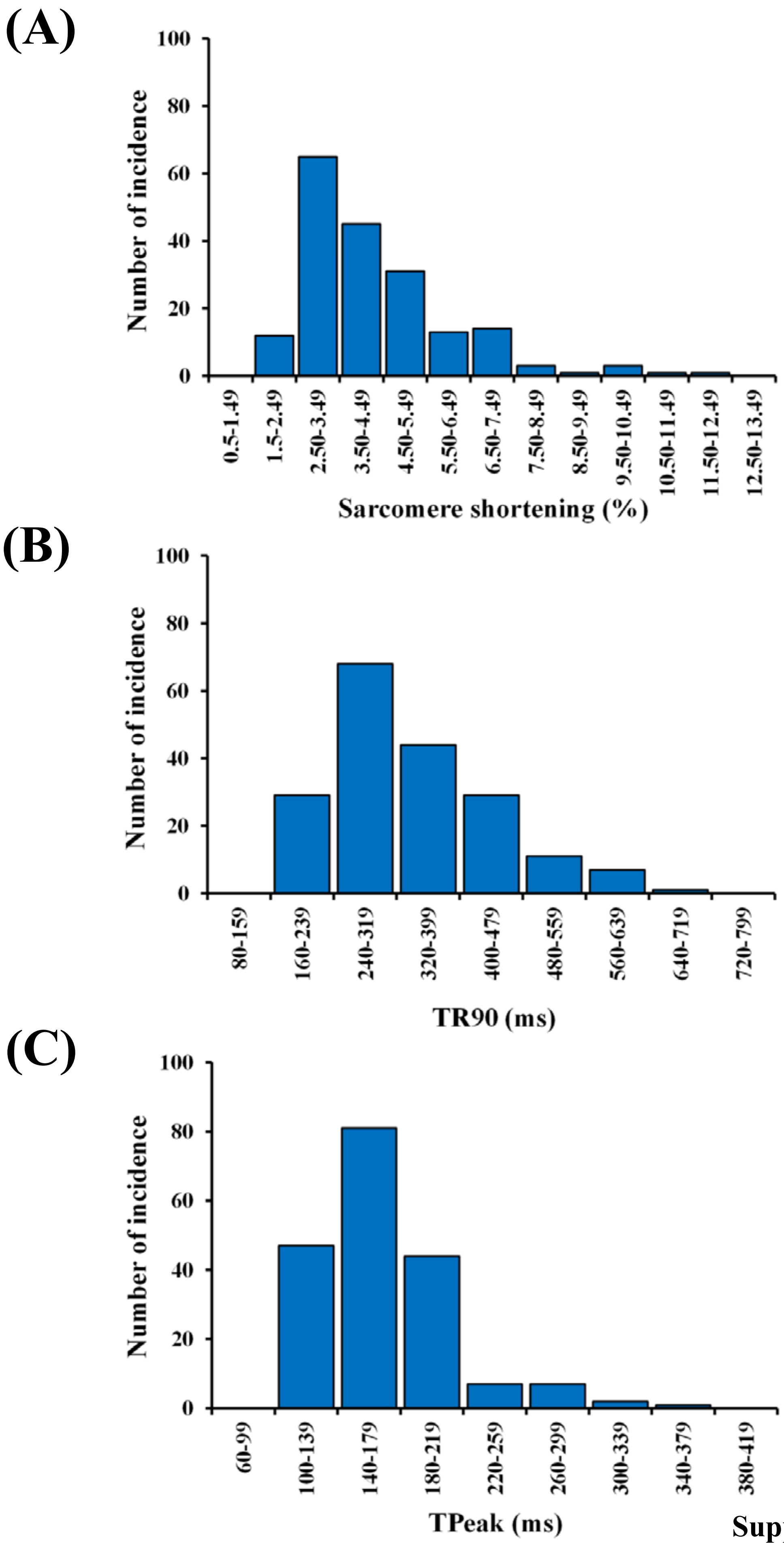

Supplementary Figure 2

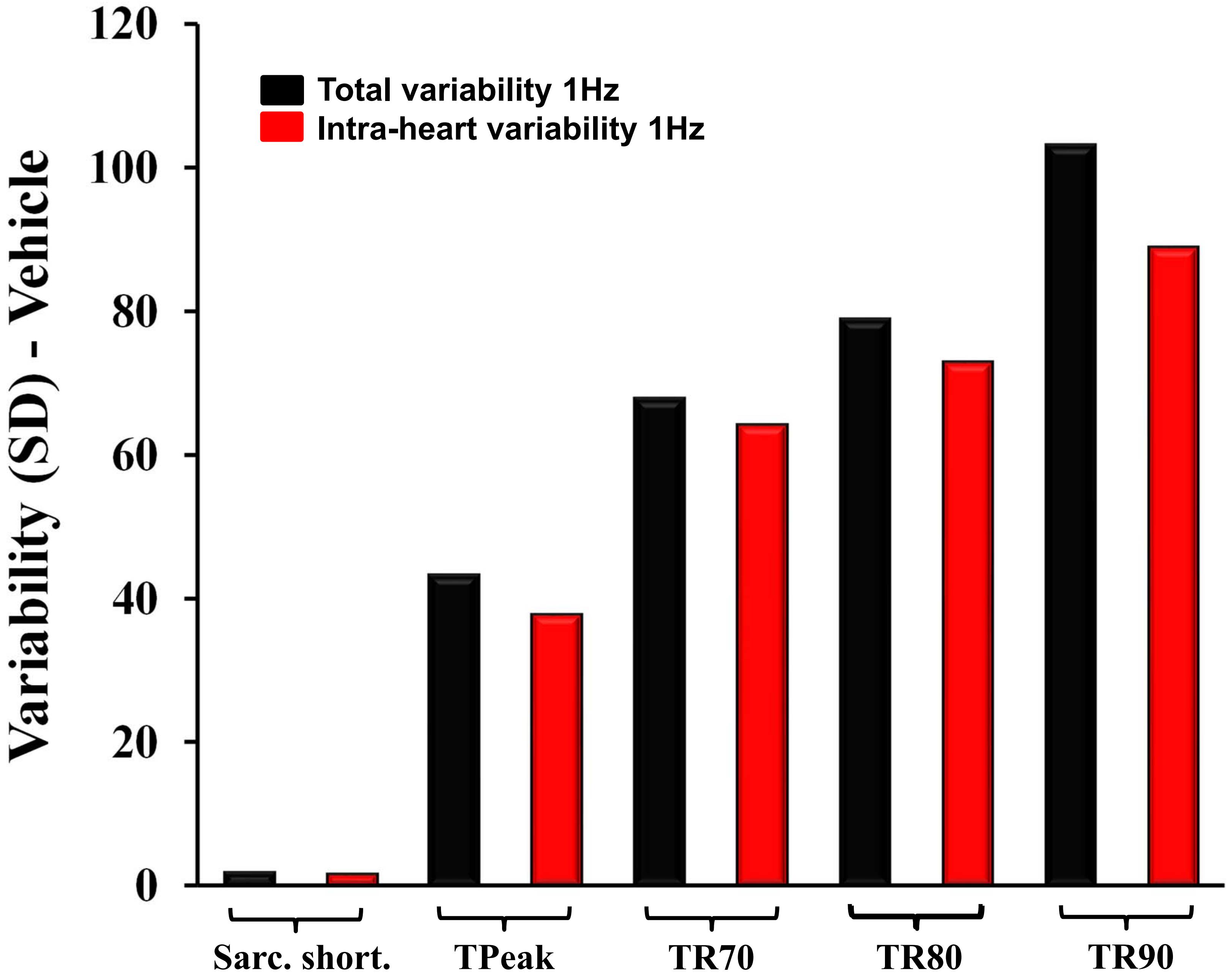

Supplementary Figure 3

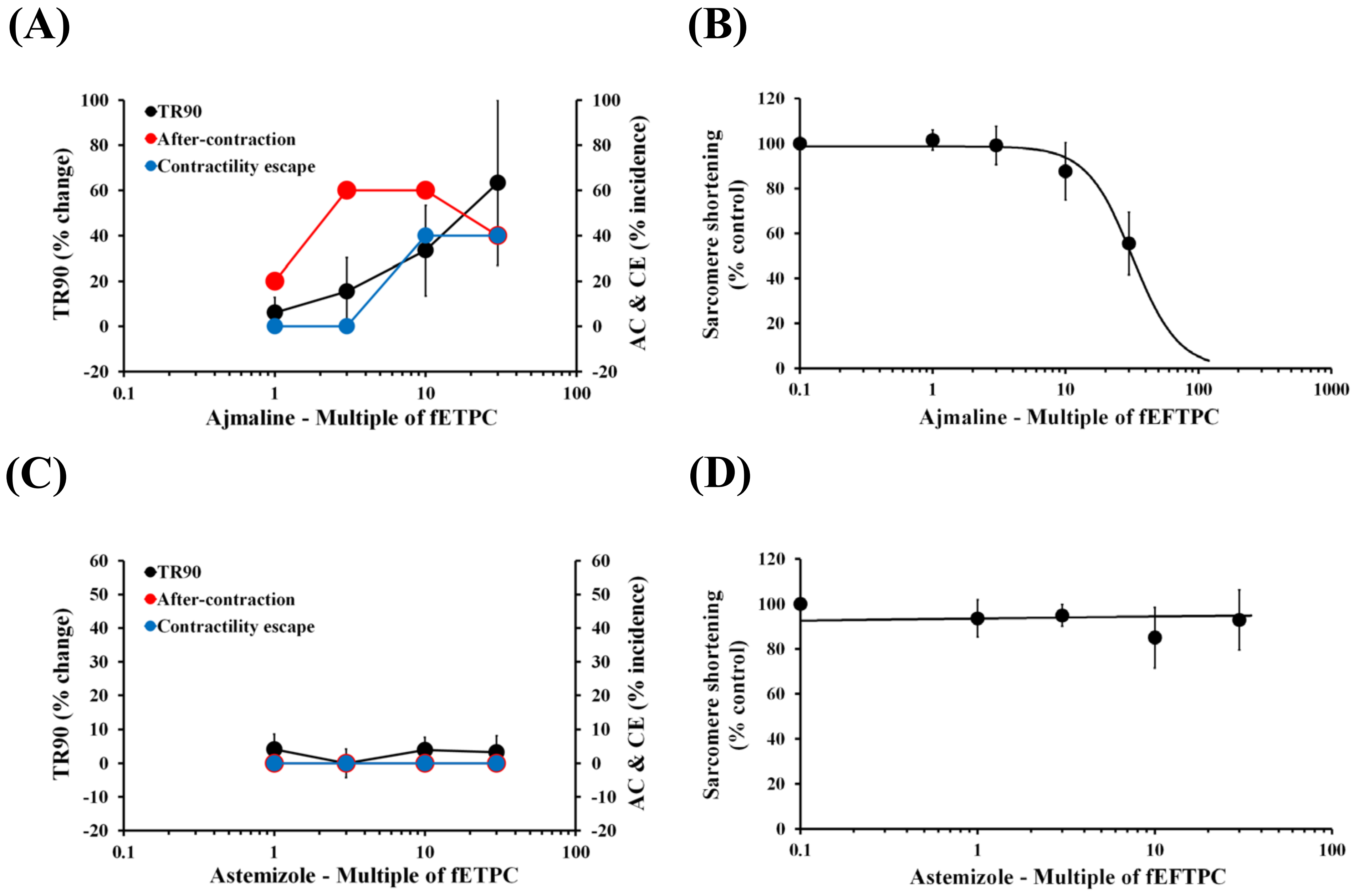

Supplementary Figure 4

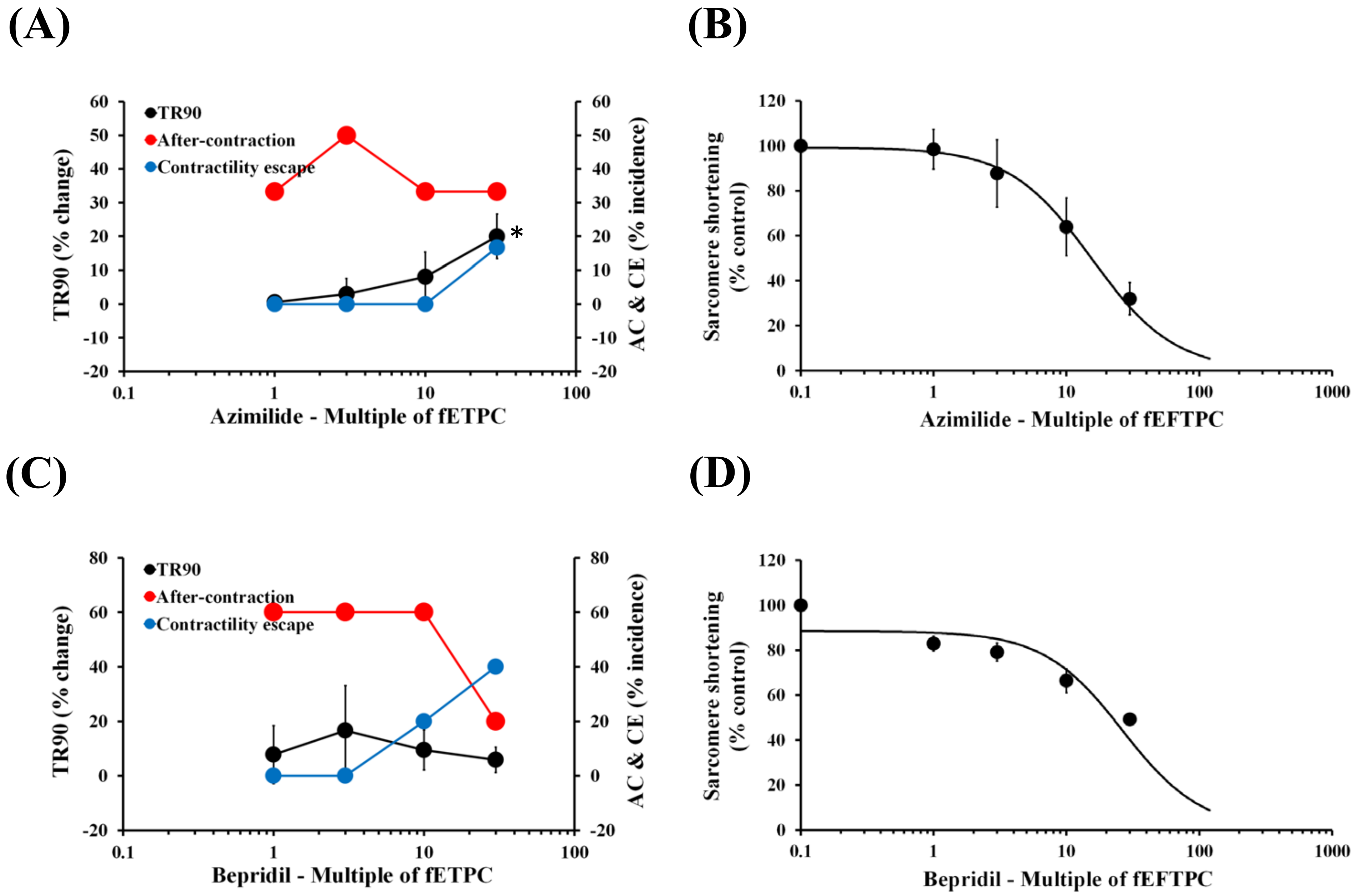

Supplementary Figure 5

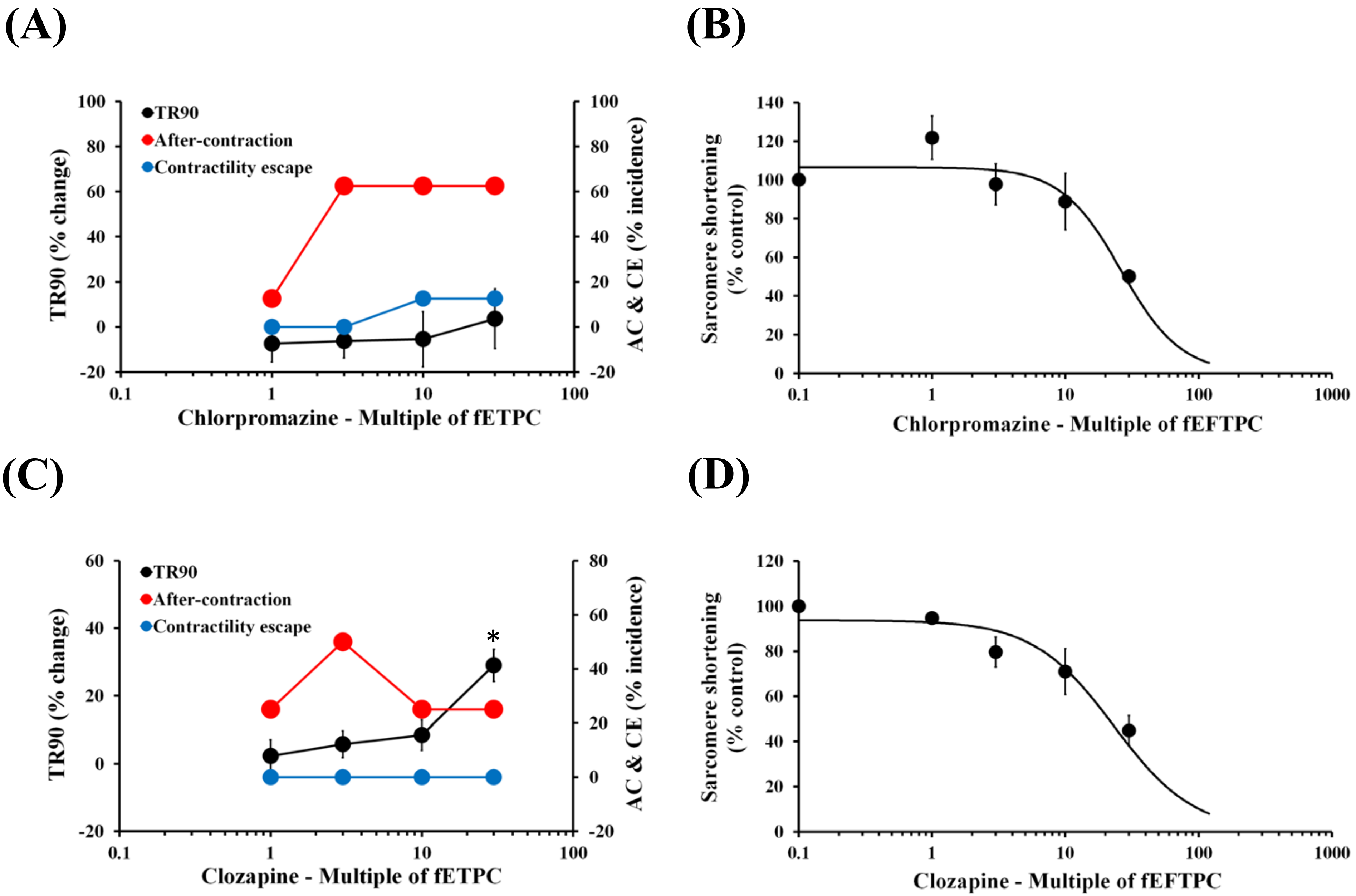

Supplementary Figure 6

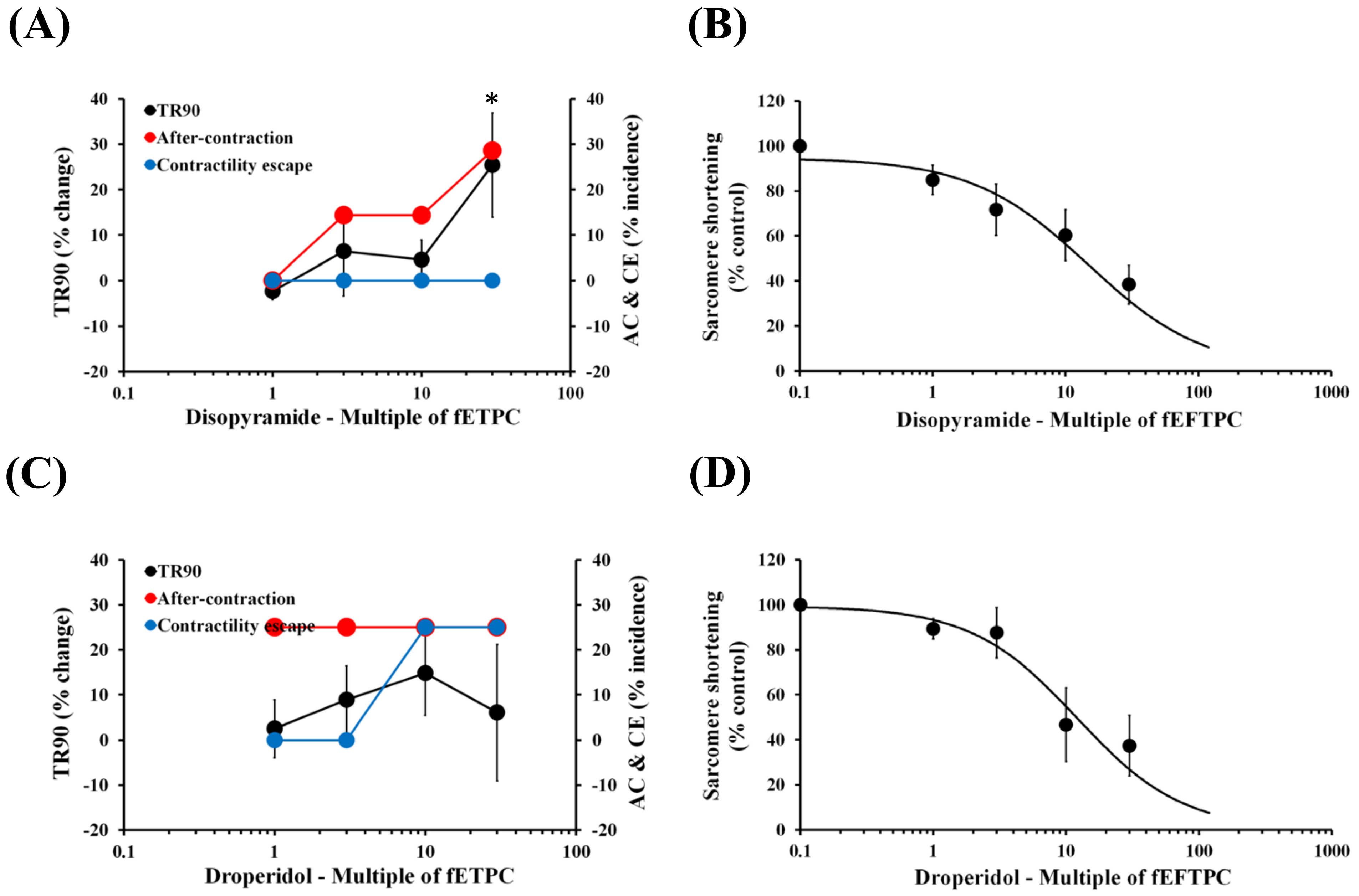

Supplementary Figure 7

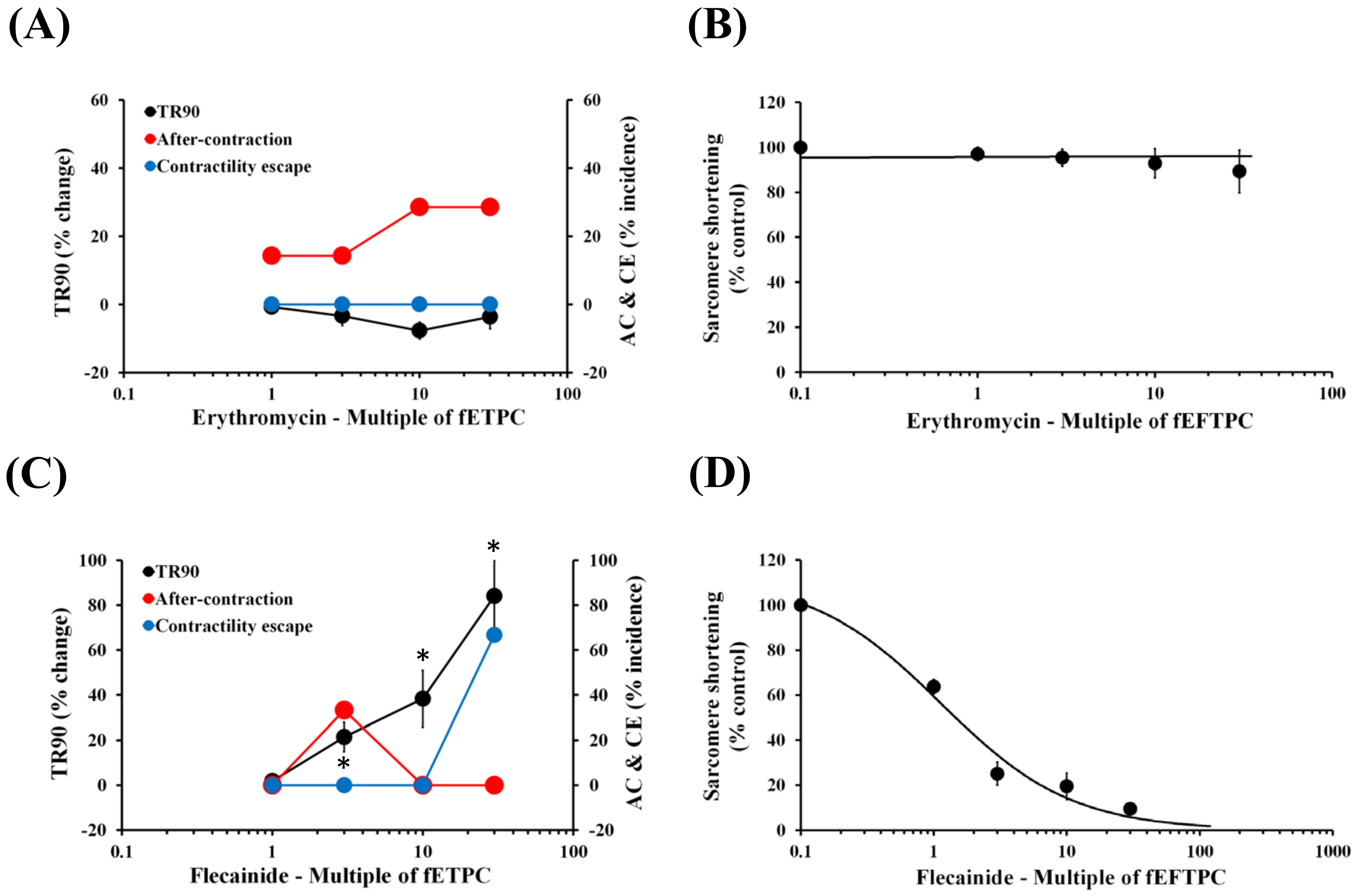

Supplementary Figure 8

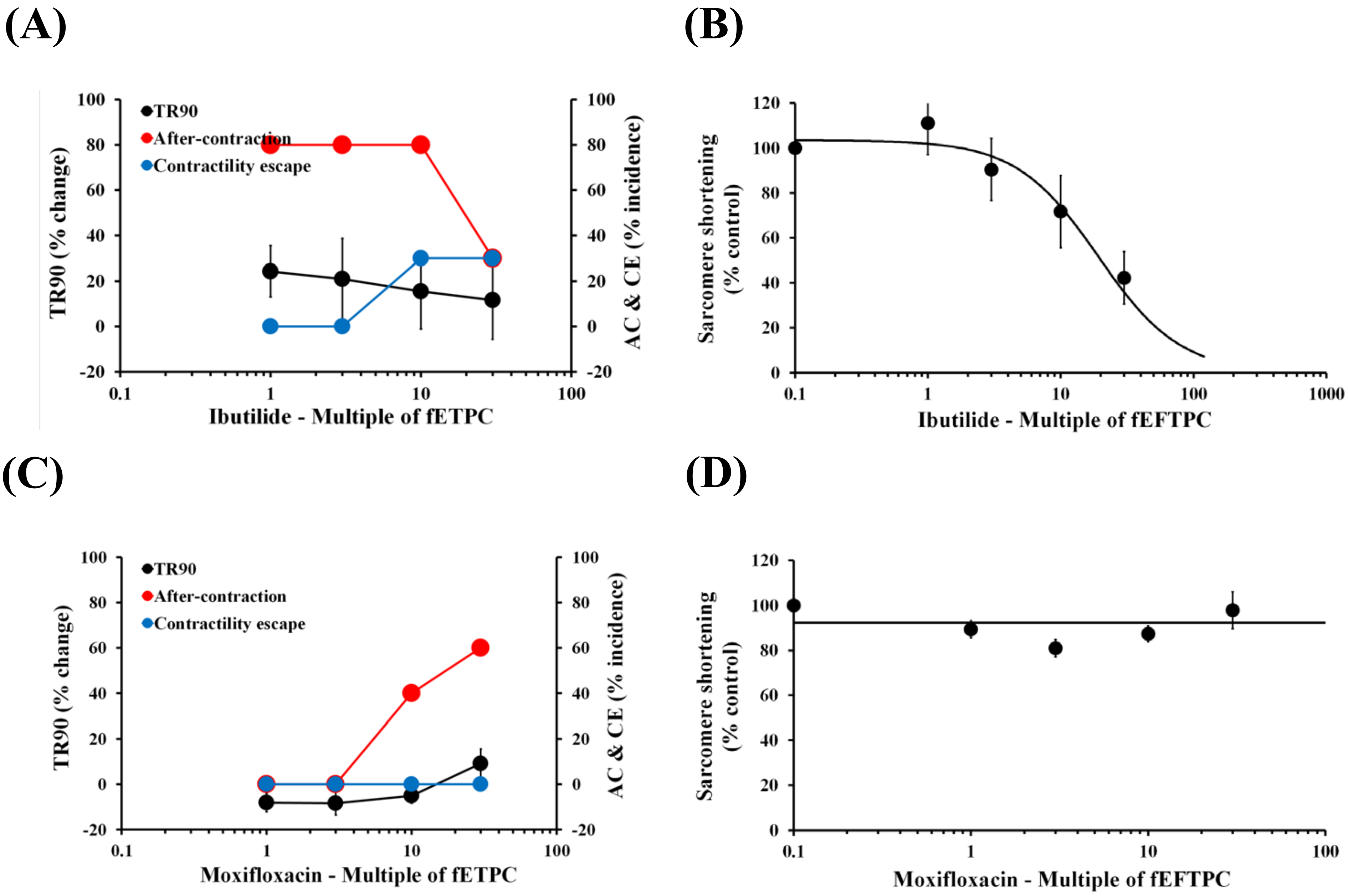

Supplementary Figure 9

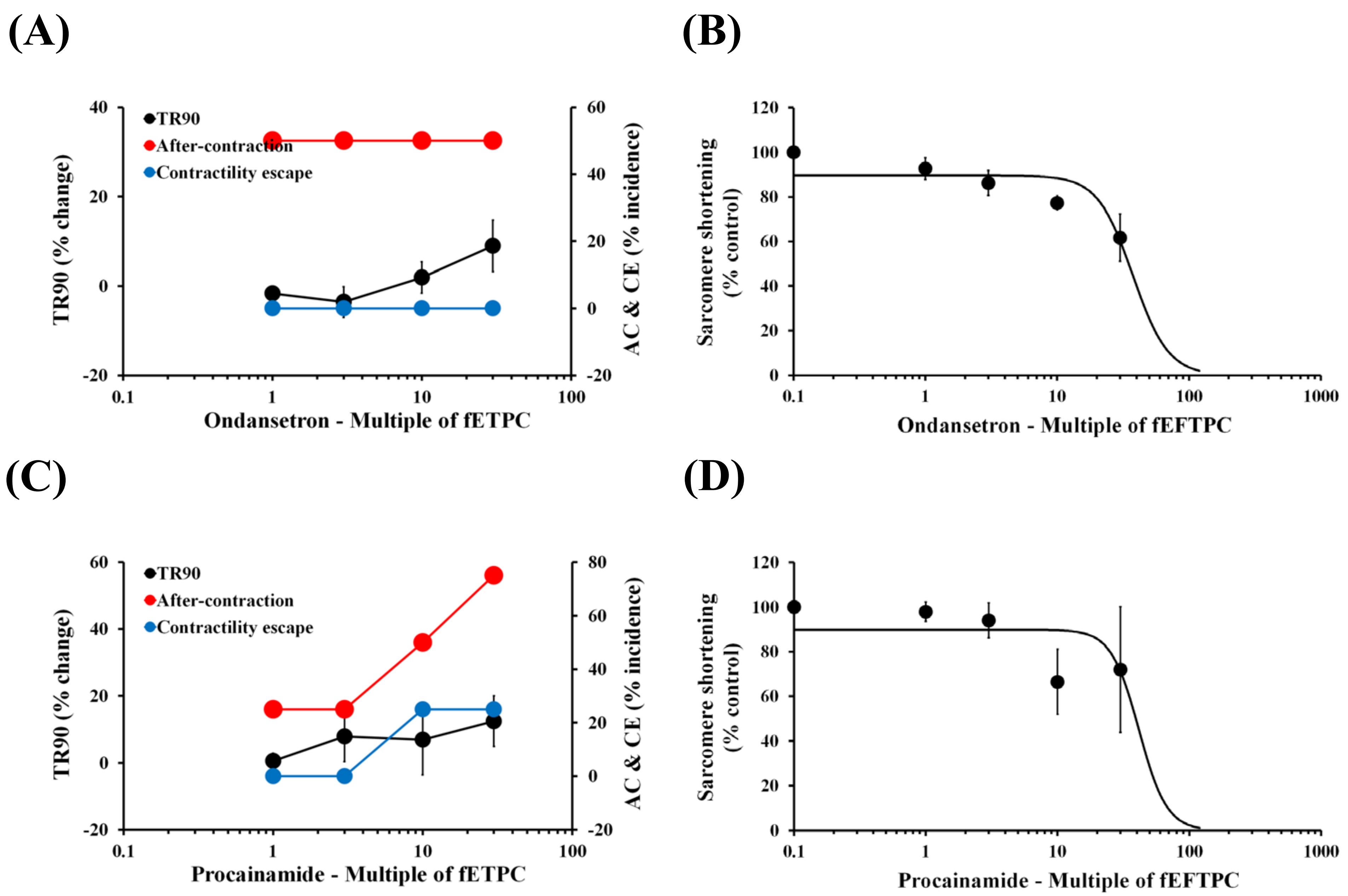

Supplementary Figure 10

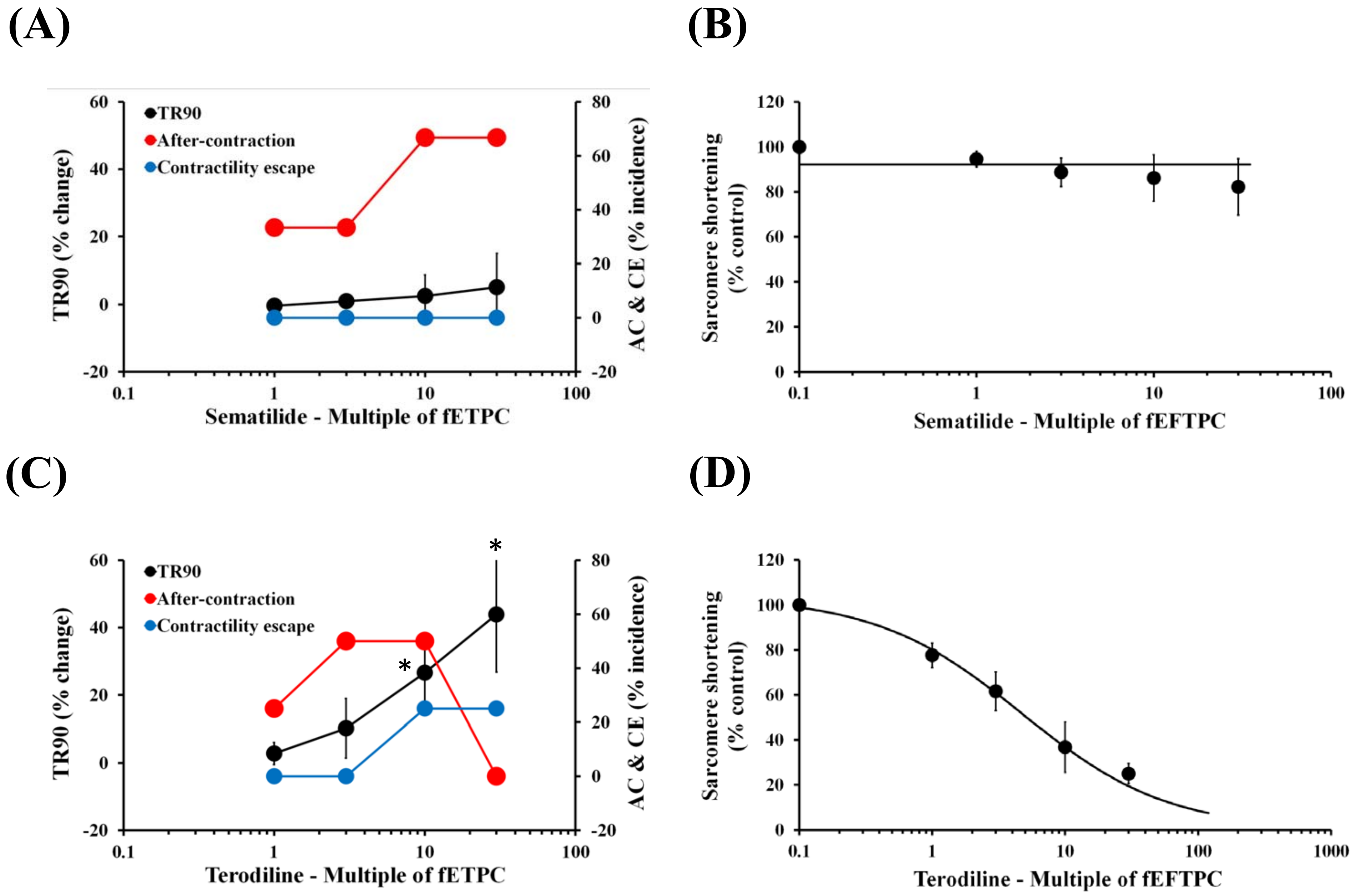

Supplementary Figure 11

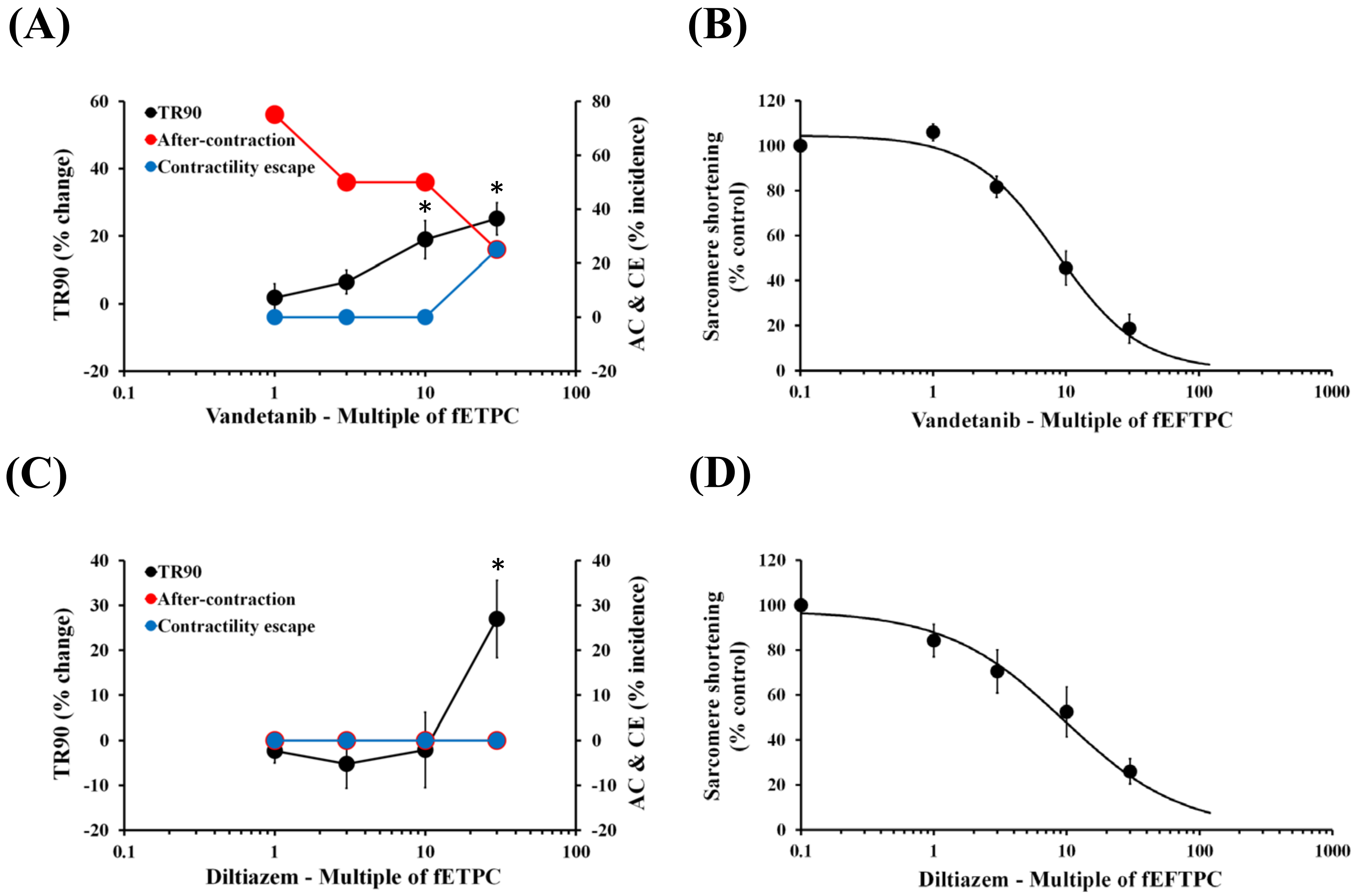

Supplementary Figure 12

(A)

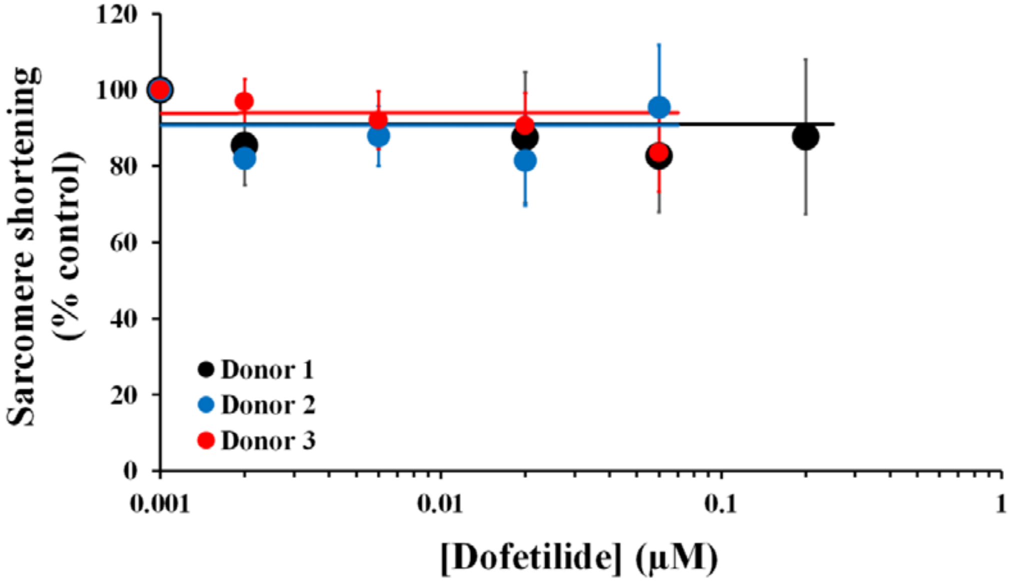

(B)

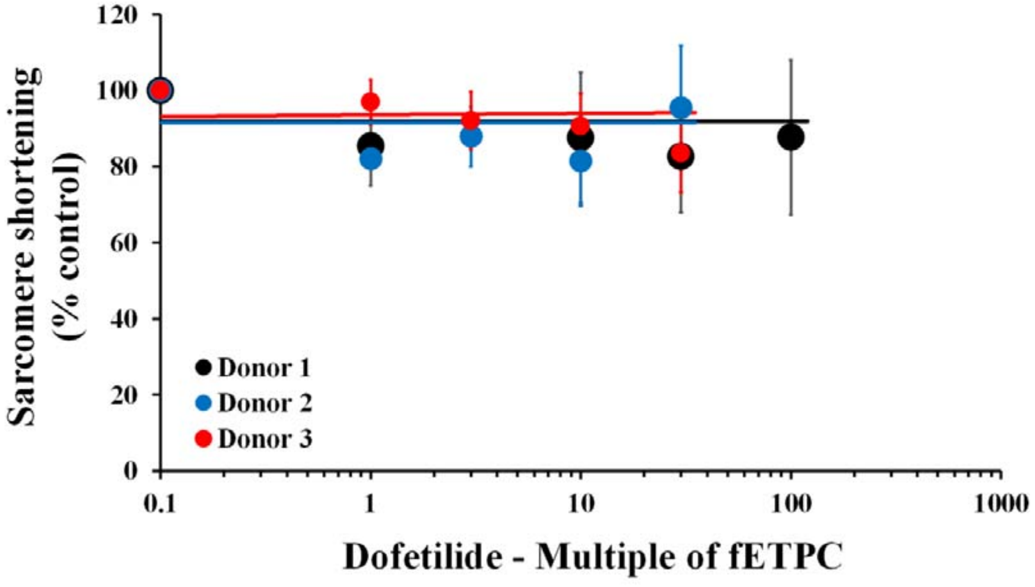

Supplementary Figure 13

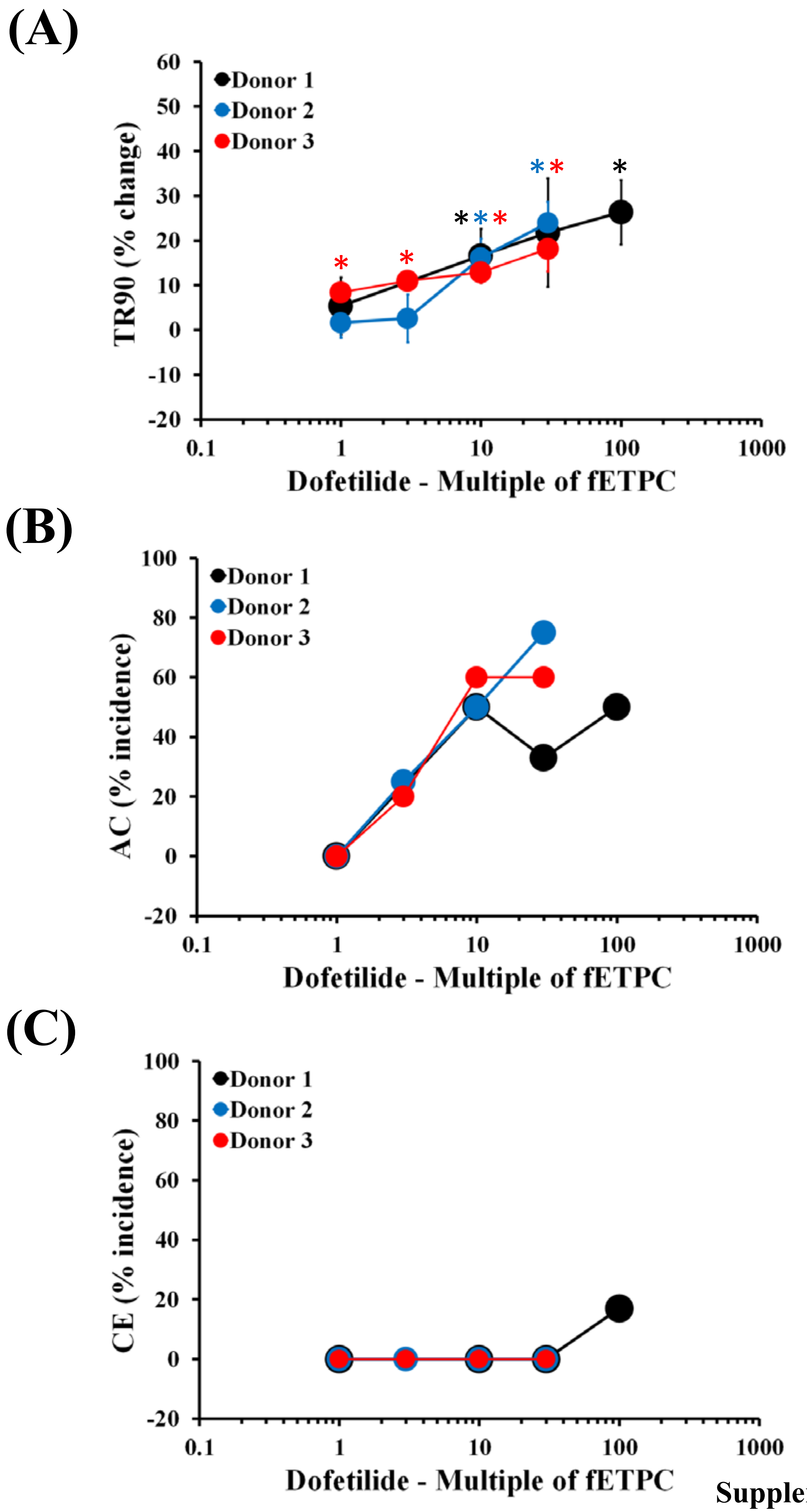

Supplementary Figure 14

(A)

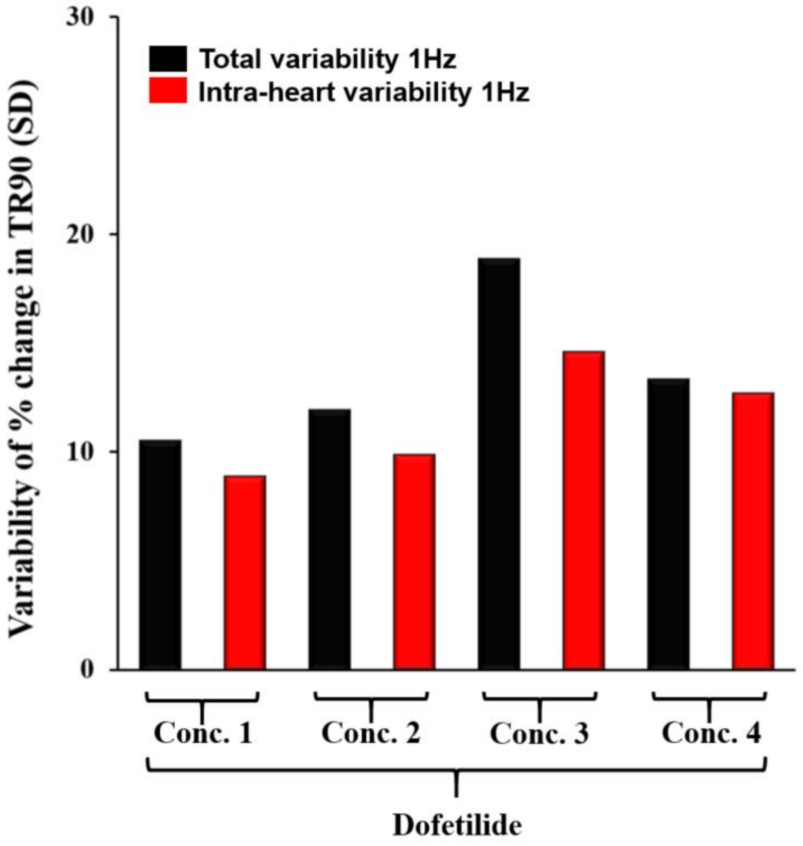

(B)

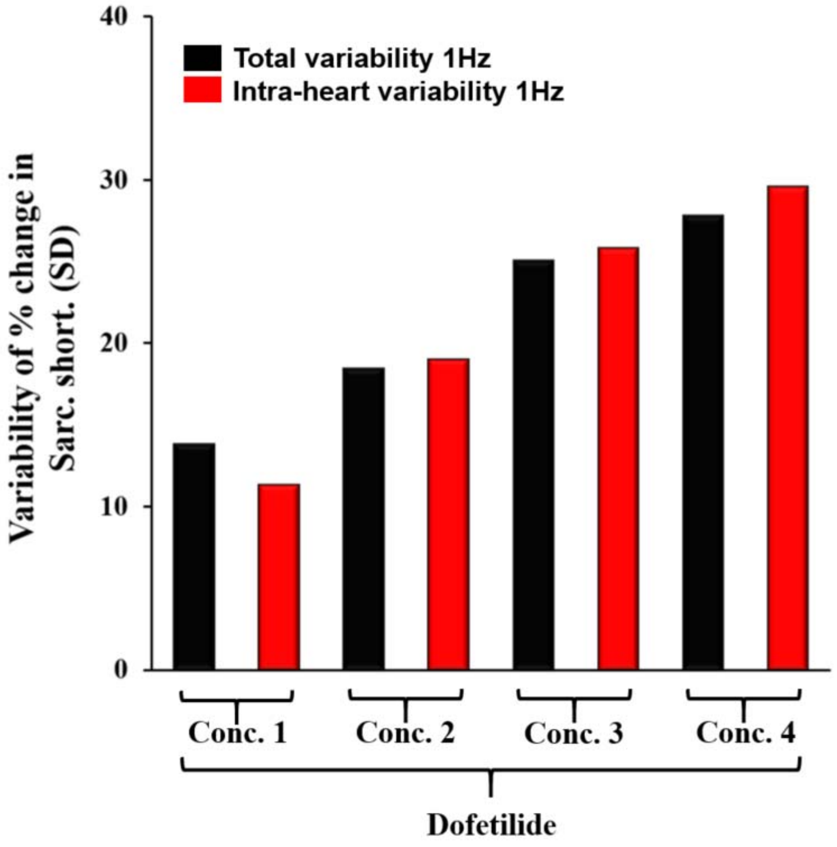

Supplementary Figure 15

(A)

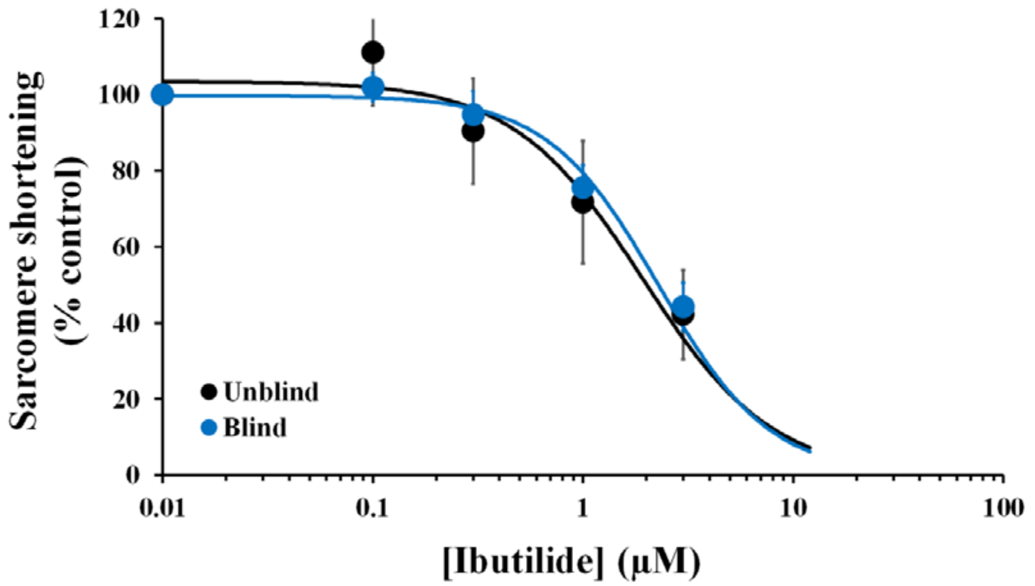

(B)

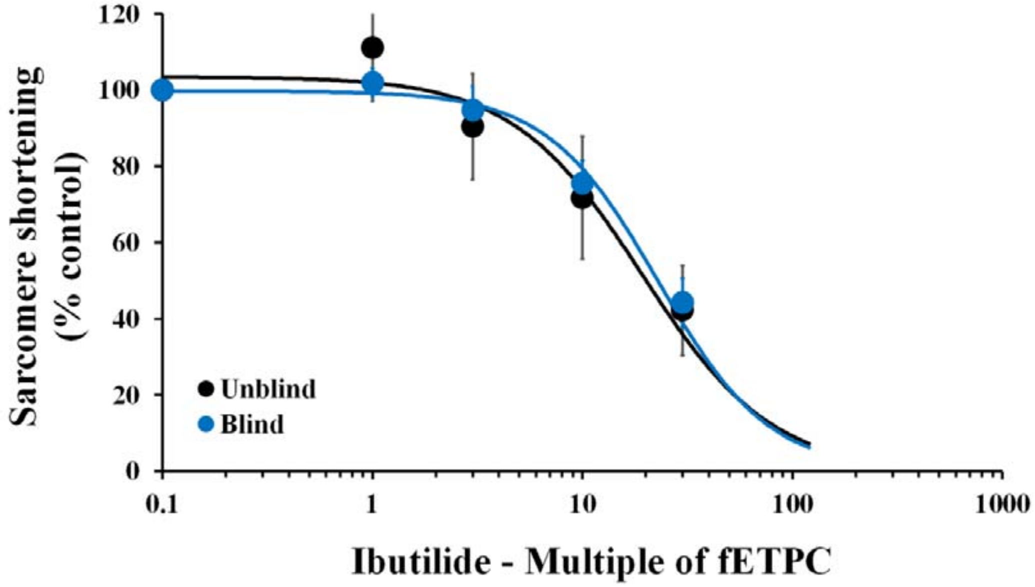

Supplementary Figure 16

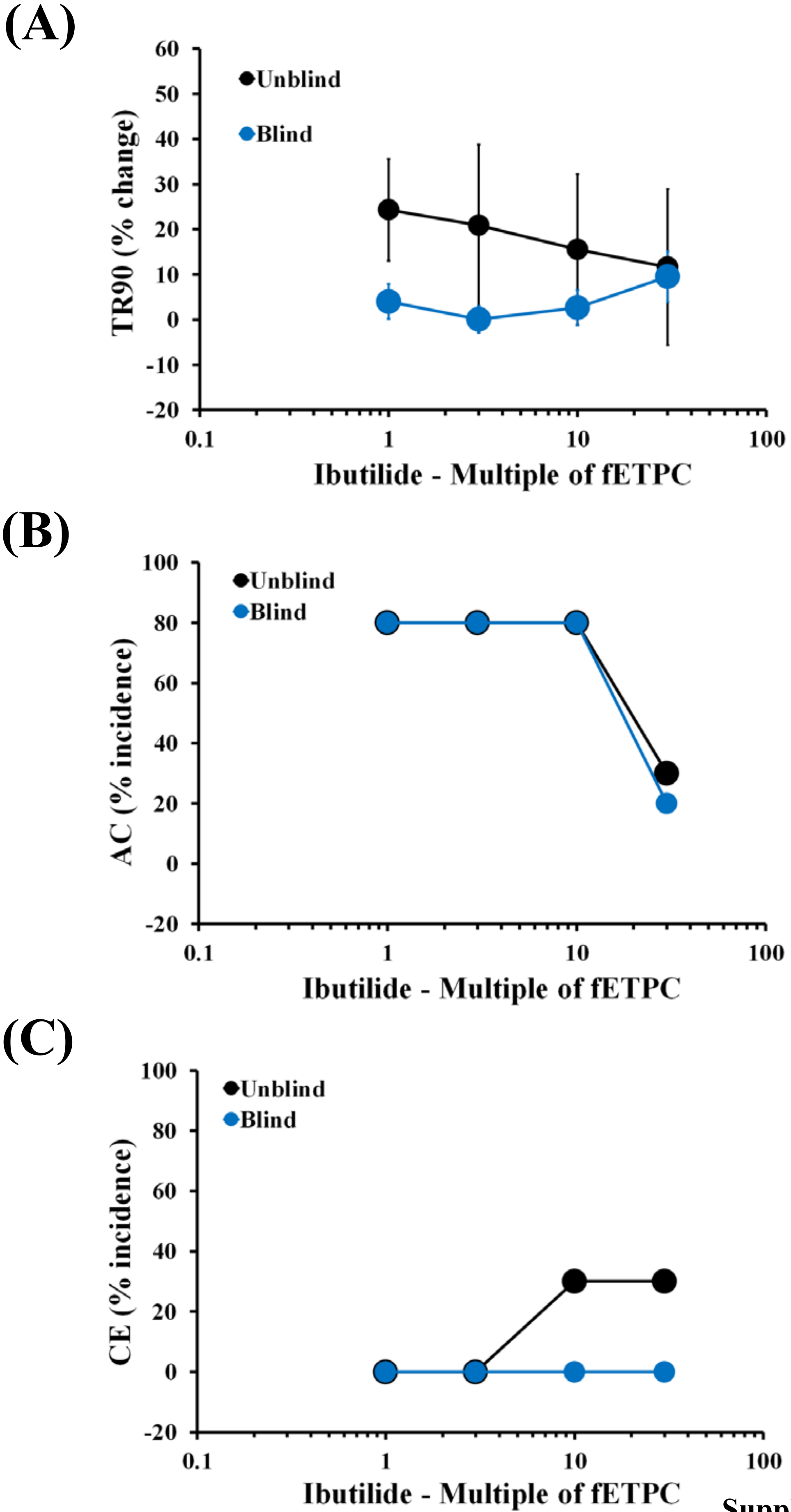

Supplementary Figure 17

(A)

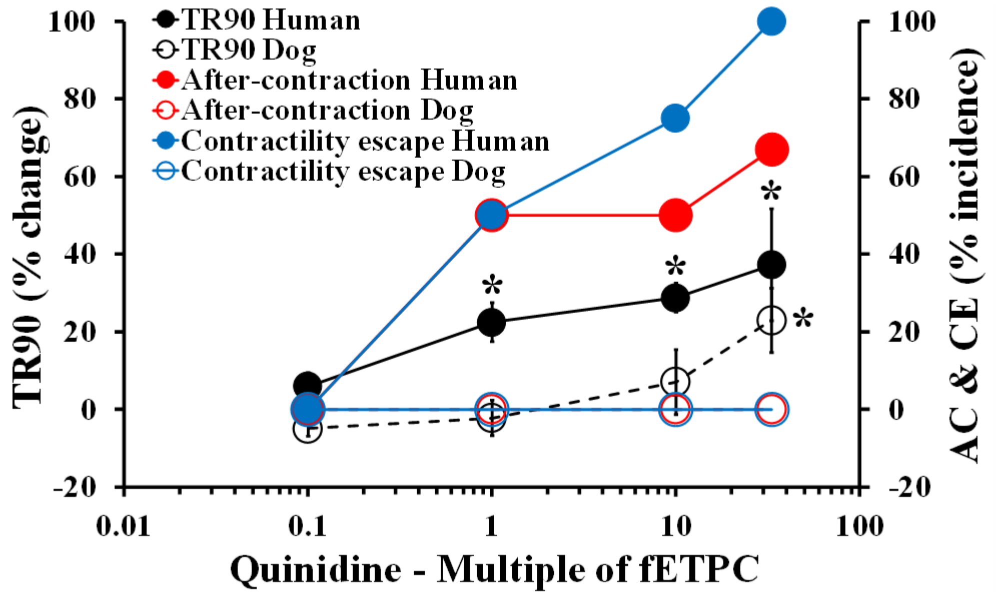

(B)

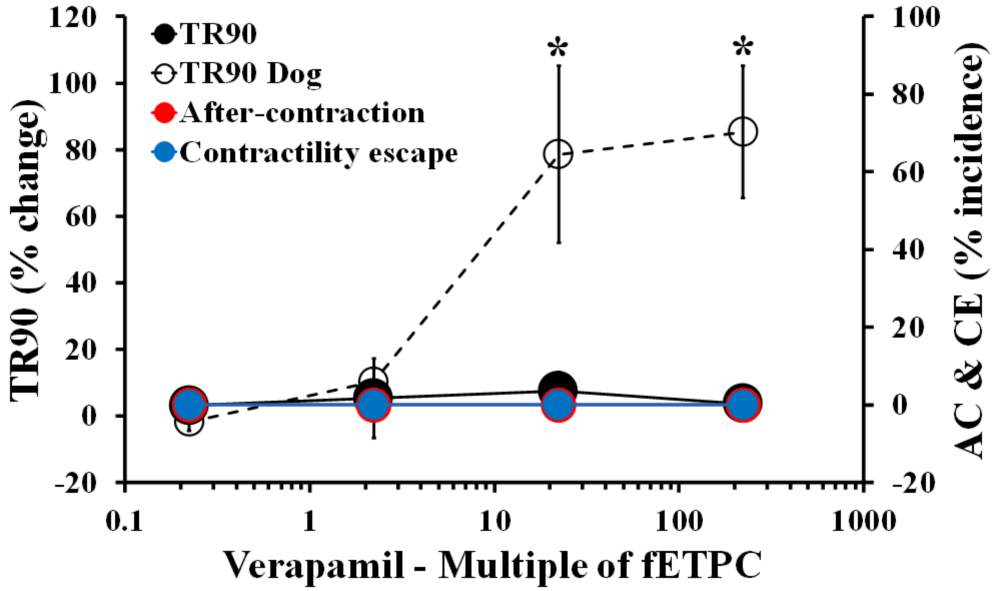

Supplementary Figure 18

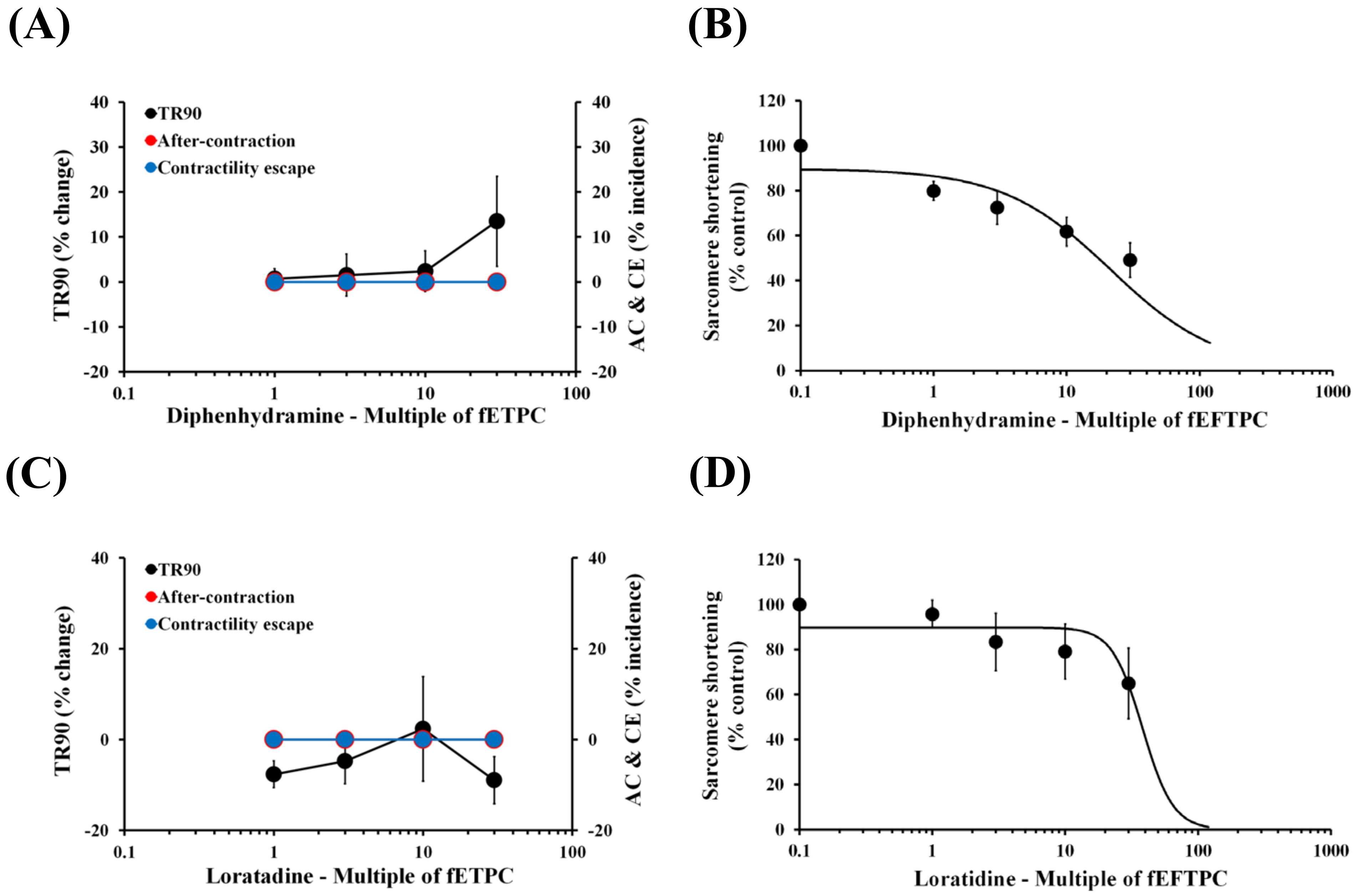

Supplementary Figure 19

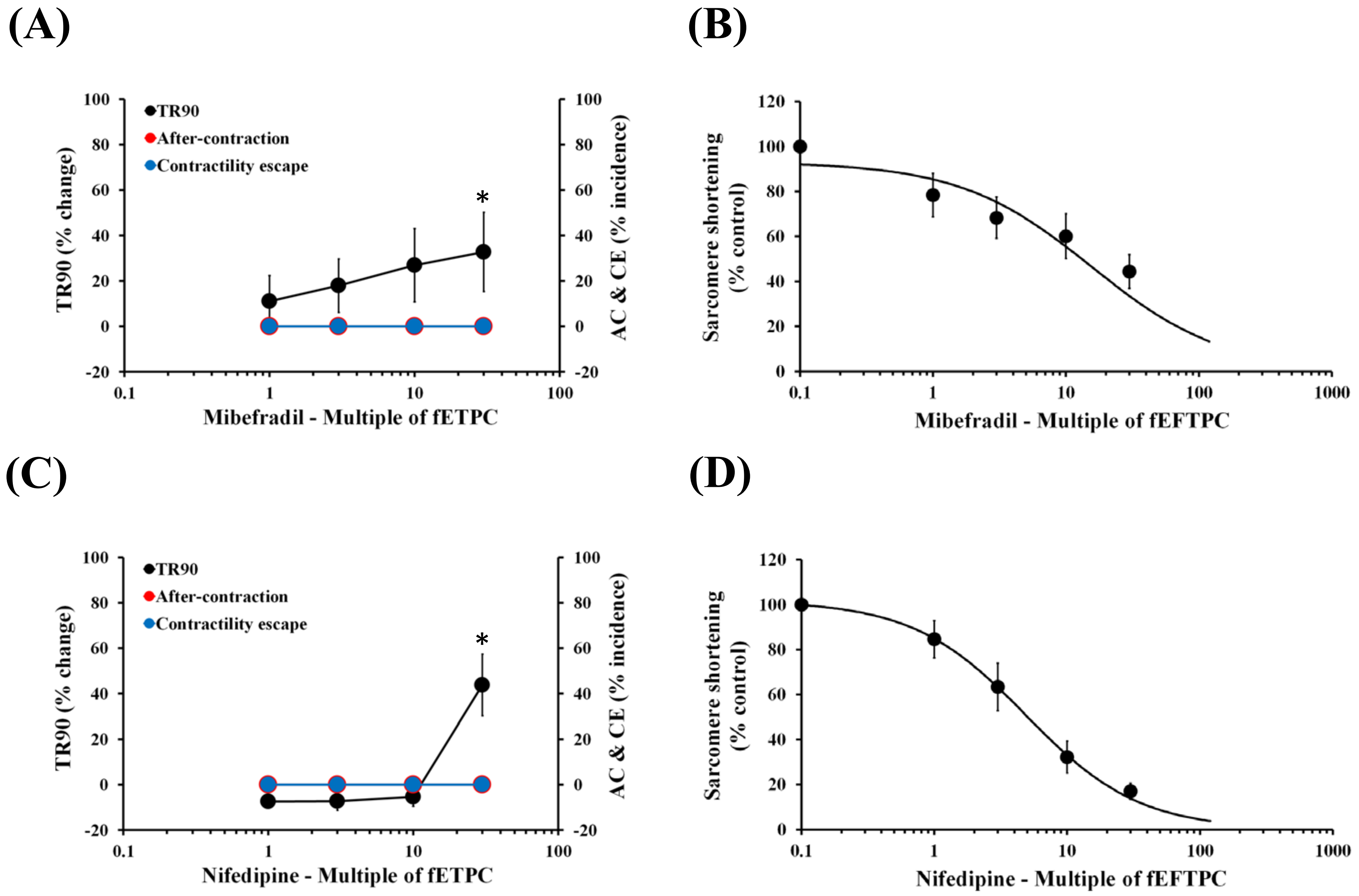

Supplementary Figure 20

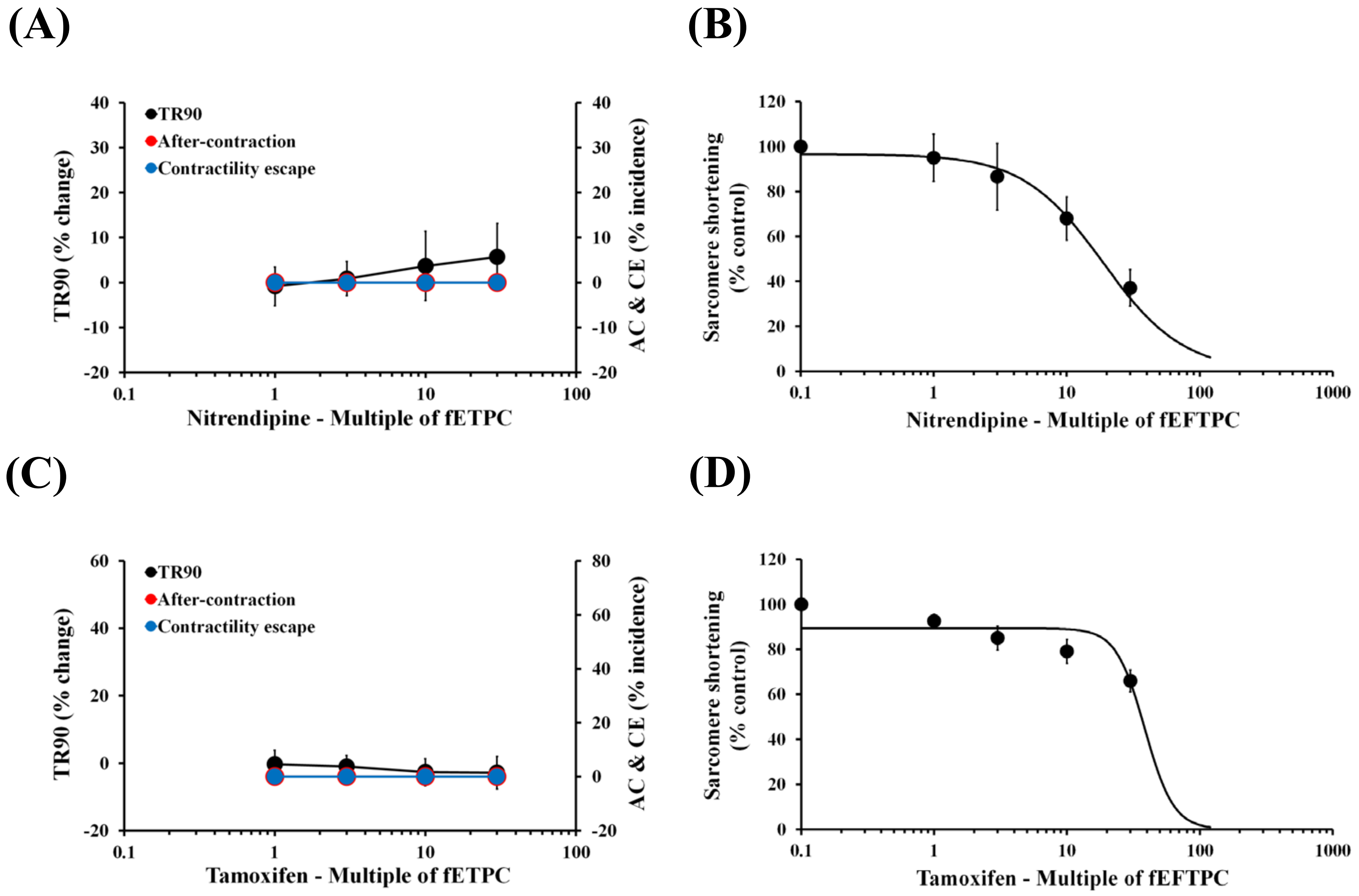

Supplementary Figure 21

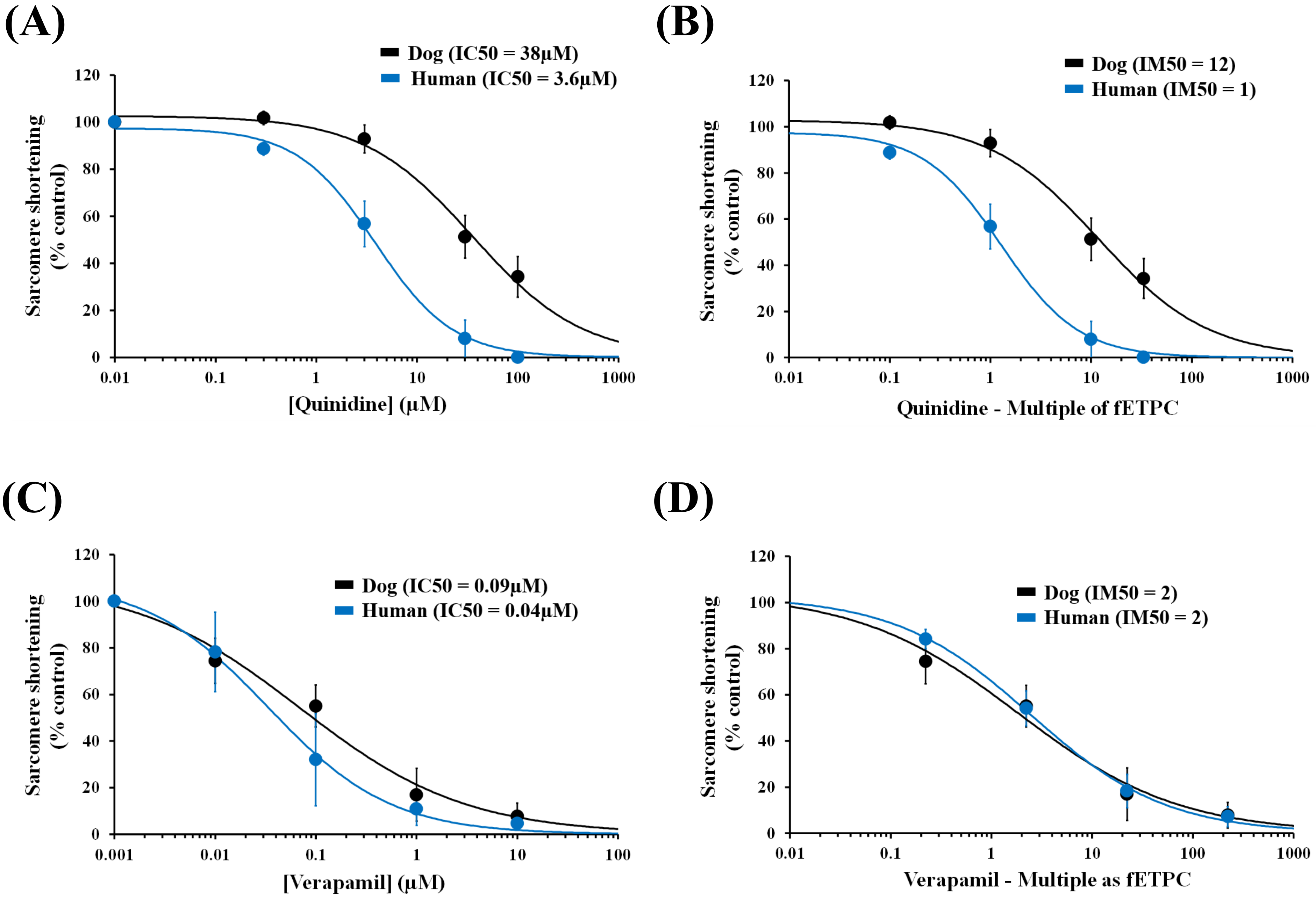

Supplementary Figure 22
